# Facile synthesis of deuterium-labeled denatonium cation and its application in the quantitative analysis of Bitrex by liquid chromatography-mass spectrometry

**DOI:** 10.1007/s00216-015-8815-y

**Published:** 2015-06-16

**Authors:** Remigiusz Bąchor, Alicja Kluczyk, Piotr Stefanowicz, Zbigniew Szewczuk

**Affiliations:** Faculty of Chemistry, University of Wrocław, F. Joliot-Curie 14, 50-383 Wrocław, Poland

**Keywords:** Bitrex, Hydrogen deuterium exchange, Isotope dilution, Quantitative analysis, Liquid chromatography-mass spectrometry

## Abstract

Quantitative analysis by liquid chromatography-mass spectrometry (LC-MS) is frequently based on the application of isotopically labeled standards which usually have to be specially synthesized. Although the synthesis of deuterated isotopologues is relatively inexpensive, they are not considered as good internal standards due to the possible deuterium effect on the retention time during LC-MS analysis. We developed a method of deuterium labeling of denatonium benzoate (Bitrex) via H/D exchange of its α-carbon hydrogen atoms in CH_2_ group situated between carbonyl and quaternary ammonium groups. The proposed strategy is rapid, cost-efficient, and does not require derivatization reagents or further purification. The LC-MS analysis of isotopologues revealed that the introduced deuterons do not undergo back exchange under acidic conditions, and the co-elution of deuterated and non-deuterated forms is observed. The obtained deuterated standard was applied in the quantitative LC-MS analysis of Bitrex in commercially available household products.

**Electronic supplementary material:**

The online version of this article (doi:10.1007/s00216-015-8815-y) contains supplementary material, which is available to authorized users.

## Introduction

Denatonium benzoate, also known as Bitrex, is a an extremely bitter compound used commonly to denature industrial alcohols and to make potential harmful household products extremely unpalatable [1]. Therefore, denatonium benzoate is an important aversive agent used in the prevention of accidental consumption of liquids such as cleaning agents, windshield washer fluids, disinfectants, and horticultural products. The Polish regulations specify that ethyl alcohol is excluded from special tax supervision while the amount of denatonium benzoate used for its denaturation is no less than 1 g per 100 dm^3^ of 100 % vol. ethanol [2].

Several methods have been applied to determine denatonium benzoate in different samples, including high-performance liquid chromatography (HPLC) [2], UV-Vis spectrophotometry [3], capillary electrophoresis [4], and liquid chromatography-atmospheric pressure chemical ionization-mass spectrometry (LC-APCI-MS) [5]. However, the proposed methods present several disadvantages; therefore, the investigation of the new analytical methodologies for the denatonium benzoate determination is required.

Recently, mass spectrometry has become a method of choice in the rapid analysis of chemicals. However, due to the low ionization efficiency of some compounds during the ESI-MS experiment, the reliable identification of trace amount of substance is often limited. It was found that quaternary ammonium (QA) group used as a fixed charge tag increases the ionization efficiency of peptides during ESI-MS analysis and significantly lowers the detection limit [6]. The QA moiety, usually based on the betaine structure, introduces a fixed positive charge to the molecule of analyte; therefore, the ionization by protonation/deprotonation or adduct formation with metal ions is not required. It is worth noting that denatonium cation contains the betaine derivative which may facilitate its sensitive analysis by ESI-MS. Additionally, the application of LC-MS has been reported for highly sensitive and selective determination of trace amounts of compounds [7, 8]. The MS-based quantification methods usually comprise the addition of defined quantities of isotopically labeled standards to the analyzed sample for the absolute quantification of compounds [9, 10]. The applied isotopological standards should exhibit chromatographic behavior identical to the native compounds, but be distinguished by their mass difference [11]. Although many different stable isotope-labeled quantification reagents have been described [1] due to their complicated and expensive synthesis, the development of new isotopically labeled standards is still under investigation.

Previously, we presented the hydrogen-deuterium exchange (HDX) of the α-C hydrogens of *N,N,N*-trialkylglycine (betaine) residue in peptides, which occurs within minutes in 1 % *N,N,N*-triethylamine (TEA) solution in D_2_O [12, 13]. We confirmed that the introduced deuterons do not undergo back exchange in acidic aqueous solutions, which may be useful in the quantitative analysis of betaine-containing compounds by LC-MS. Additionally, we found that α-C hydrogens of *N*-methylglycine (sarcosine) residue undergo H/D exchange in 1 % TEA/D_2_O solution; however, this process is relatively slow [14]. We have also investigated the HDX reaction at the histidine C2 atom of imidazole in phosphorylated histidine [15]. Additionally, the growing importance of H/D exchange reaction and their applicability in the chemical analysis was widely presented by Atzrodt and co-workers [16].

In this article, we present our studies on synthesis of the α-C deuterated analogue of denatonium cation containing betaine derivative and its application in quantitative analysis of denatonium benzoate containing samples by LC-MS.

## Results and discussion

**Fig. 1:**
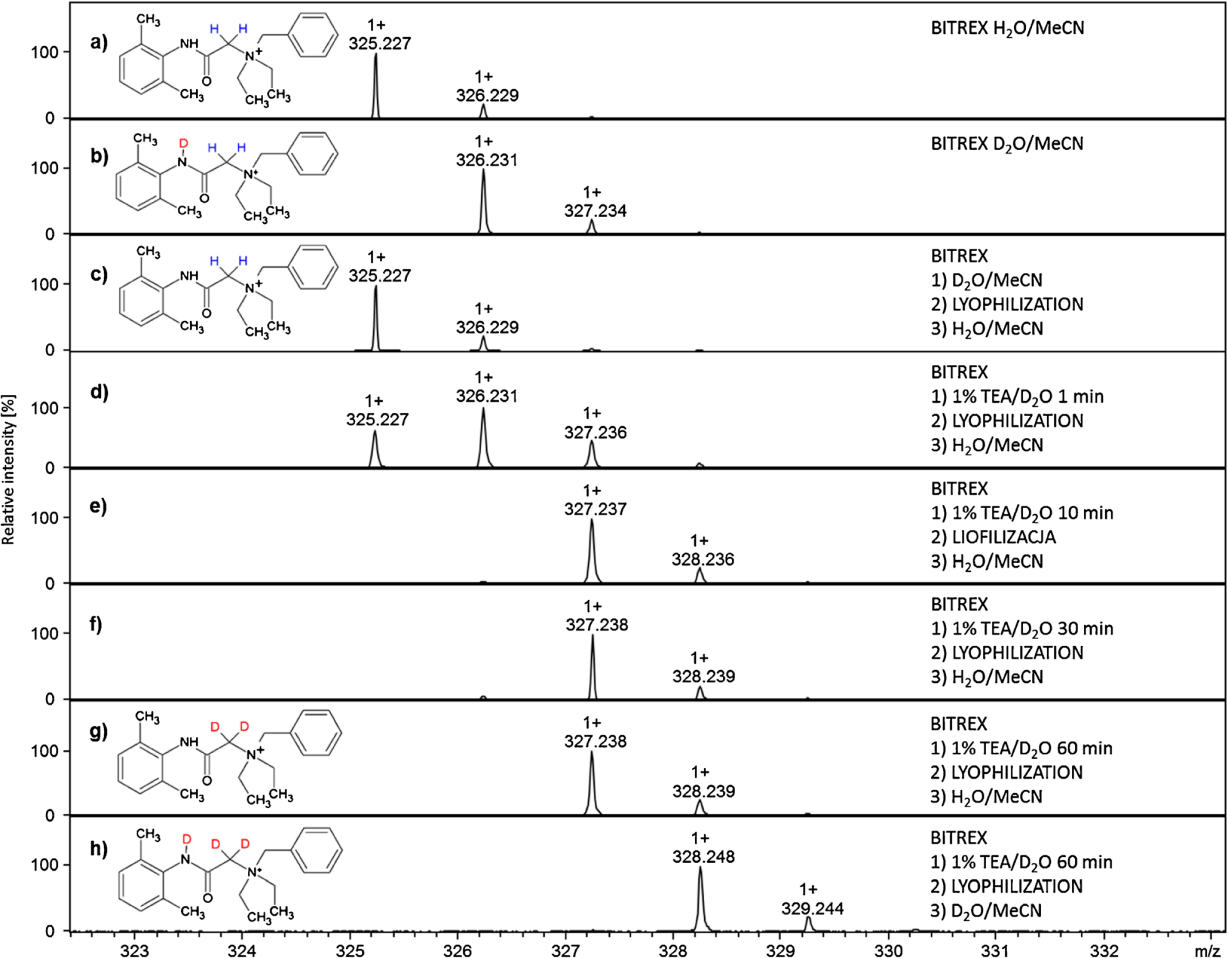
ESI-MS spectra in positive ion mode of denatonium benzoate dissolved in: **a** acetonitrile–water mixture containing 0.1 % HCOOH; **b** D_2_O; **c** after back exchange of amide deuteron; and after incubation in 1 % TEA/D_2_O for **d** 1 min **e** 10 min; **f** 30 min; **g** 60 min, lyophilization and sample redissolving in acetonitrile–water mixture containing 0.1 % HCOOH; **h** 60 min incubation in 1 % TEA/D_2_O, lyophilization and sample redissolving in D_2_O

The aim of this work was to obtain the α-C deuterated analogue of denatonium cation and to analyze their applicability in quantitative analysis of denatonium benzoate containing samples by LC-MS. Denatonium cation is a derivative of betaine, which was found by us previously to undergo HDX at the α carbon under basic conditions within few minutes at room temperature [12]. Therefore, to perform the HDX at the α-C of denatonium cation, a denatonium benzoate sample was dissolved in 1 % TEA/D_2_O solution and incubated from 1 min to 4 h at room temperature. Then, samples were lyophilized and redissolved in acetonitrile–water mixture containing 0.1 % HCOOH. According to our previous study [12], under such conditions, the deuterons introduced at the α-C of betaine derivatives do not undergo back exchange, which allows the identification of deuterated denatonium cation. The progress of HDX was investigated by ESI-MS (Fig. 1).

The obtained ESI-MS spectra (Fig. 1) show characteristic changes in isotopic distribution of signal corresponding to the denatonium cation (*m/z* 325.277) after incubation under basic conditions. It was found that in D_2_O, only the amide hydrogen was replaced by deuteron which caused the mass shift of 1 Da (*m/z* 326.231, Fig. 1b). The addition of TEA catalyzed the hydrogen–deuterium exchange at the α-C in betaine derivative, observed even after a 10-min reaction (Fig. 1e). The most intensive signal (*m/z* 327.238) corresponds to the doubly deuterated denatonium cation; however, according to the low-intensity signal at *m/z* 326.231, a small amount of unexchanged fraction still remains. The complete deuteration of α-C was observed after 60 min of incubation in 1 % TEA/D_2_O (Fig. 1g), which was additionally confirmed by ^1^H NMR analysis. The complete ^1^H NMR spectra are presented in the Electronic Supplementary Material (ESM; Fig. S1–S3).

The possibility of back exchange of deuterons introduced at the α-C was also investigated. The sample of α-C deuterated denatonium benzoate was dissolved in H_2_O. It was found that under such reaction conditions, the back exchange of introduced deuterons does not occur even after a 2-week incubation at room temperature. Only the addition of 1 % TEA (pH = 11.9) results in the rapid exchange of the α-C deuterons into hydrogens. The kinetics of this reaction was analyzed by ESI-MS, and the total back exchange was confirmed by ^1^H NMR (see ESM, Figs. S4, S5). Additionally, the stability of denatonium cation under HDX reaction conditions was analyzed by ^1^H NMR, and the obtained results revealed that the decomposition does not occur (see ESM, Figs. S6–S9).

The above results revealed that the incubation of denatonium benzoate in 1 % TEA/D_2_O solution results in the complete HDX at the α-C of denatonium cation. This suggests the possible application of proposed HDX method as a rapid, simple, and cost-efficient strategy for the preparation of deuterated denatonium cation as compared to its de novo synthesis. The deuterated standard may be useful in the quantitative LC-MS analysis of denatonium benzoate containing samples.

The effect of isotopic labeling of the α-C of amino acid residues and their derivatives on chromatographic separations has been previously described in the literature [17, 18]. It was mentioned that stable isotopes, like ^13^C, ^15^N, and ^18^O, practically do not affect the retention, although in the case of ^2^H, a significant isotopic effect was observed [19, 20]. Therefore, in many cases, the deuterated analogues are not considered as appropriate internal standards in isotopic dilution mass spectrometry for quantitative analysis. The difference in the retention time of isotopologues containing deuterons significantly hinders their quantitative analysis by LC-MS. Additionally, it was mentioned by Zhang an co-workers [20] that the introduction of deuterons closely to the polar center has little or no effect on the retention of isotopologues. Therefore, it may be speculated that deuterons introduced at the α carbon in the betaine derivative of denatonium cation may not affect the co-elution of labeled and non-labeled compounds.

**Fig. 2:**
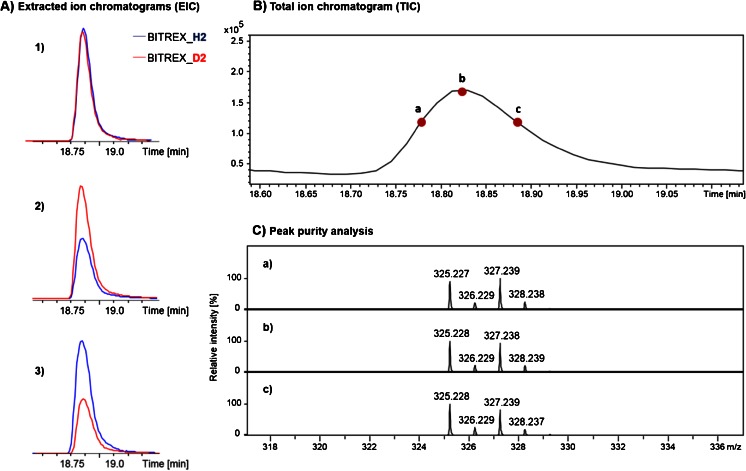
Extracted ion chromatograms (**A**) of deuterated and non-deuterated denatonium benzoate samples mixed in different ratios: (*1*) 1:1, (*2*) 2:1, and (*3*) 1:2. The total ion chromatogram peak at Rt 18.83 min was selected for peak purity analysis (**B**). The ESI-MS spectra presented for points *a*–*c* of the total ion chromatogram (**C**)

To analyze the deuterium effect on the chromatographic separation of denatonium benzoate isotopologues, the LC-MS analysis was performed. The extracted ion chromatograms revealed that the retention times of deuterated and non-deuterated denatonium cations are practically identical during the chromatographic separation for samples containing different concentrations of deuterated standard (Fig. 2a). To confirm the observed lack of isotopic effect, the peak purity analysis for a selected peak (Rt 18.83 min, Fig. 2b) of the total ion chromatogram (TIC) was performed. The ESI-MS spectra obtained for the different points on TIC present similar isotopic patterns, indicating the consistent amounts of the two isotopologues, and thus their co-elution (Fig. 2c).

The applicability of the obtained deuterated analogues of the denatonium cation in the quantitative LC-MS analysis of denatonium benzoate containing samples was investigated. Four different commercially available denatonium benzoate containing samples, including alcoholic thinner and winter/summer windscreen washer fluids, were analyzed. The different volumes of alcoholic thinner (1300, 1000, 700, 500, 200, 50, 30, and 20 μL) were evaporated, and the residues were dissolved in H_2_O (200 μL) before LC-MS analysis. A 20-μL aliquot of such solution was diluted with H_2_O (123 μL), and then 2 μL of deuterated denatonium benzoate solution in H_2_O (0.5 mg/mL) was added. The obtained extracted chromatograms for deuterated and non-deuterated denatonium cations are presented in the ESM (Fig. S10). The level of detected denatonium cations in the prepared samples was determined by the isotopic distribution observed in the ESI-MS spectra according to the algorithm described by Mirgorodskaya and co-workers [21]. The results of computational analysis are presented in the ESM (Figs. S11–S18, S22, S23). The observed isotopic patterns in experimental ESI-MS spectra are closely related to those presented in the mass spectra resulting from computational analysis, which suggests the possibility of application of such calculations in quantitative analysis of denatonium cation.

**Fig. 3:**
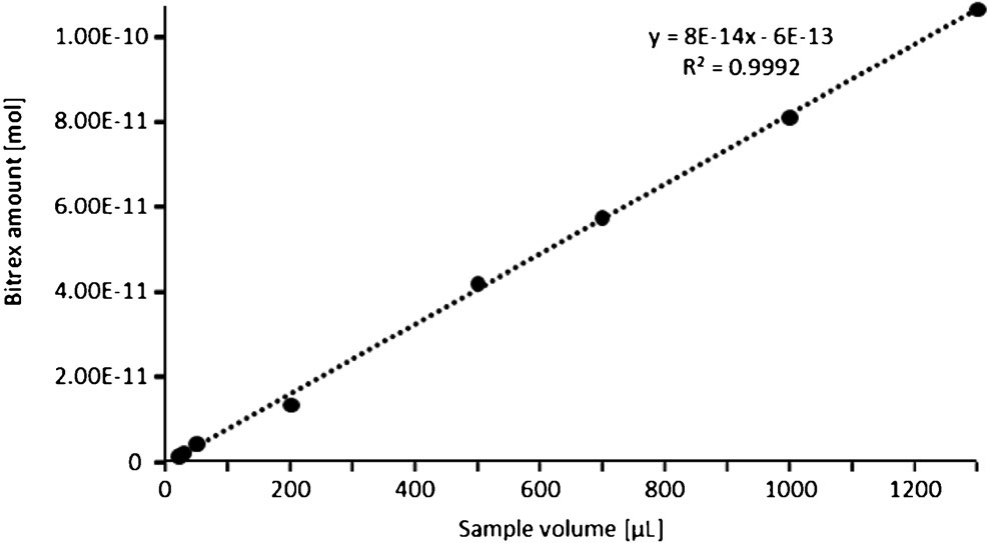
The correlation between the denatonium benzoate concentration and the amount of analyzed sample obtained by the LC-MS analysis of alcoholic thinner

The performed LC-MS analysis of alcoholic thinner samples was used to investigate the correlation between the denatonium benzoate concentration and the amount of analyzed sample for denatonium benzoate in the range from 0.68 to 47.5 ng (Fig. 3). The concentration response relation was linear (*R*^2^ = 0.9992) within this range. The calculated participation of deuterated standard and non-deuterated denatonium cation present in the analyzed samples allowed us to determine the limit of detection which was estimated as 0.68 ng (1.52 pmol) of denatonium benzoate, using standard, commercially available ESI-TOF apparatus. However, it can be expected that application of nano LC-MS operated in the multiple reaction monitoring mode may further increase the sensitivity of detection [8].

The LC-MS analysis of denatonium benzoate in the winter/summer windscreen washer fluids was also performed. The analyzed samples are complex mixtures containing ethyl and methyl alcohols, glycols, surfactants, several fragrances, and denatonium benzoate. The samples were prepared according to the procedure presented in the ESM. The obtained results presented in the ESM (Figs. S28–S33) confirmed the applicability of the proposed methodology of quantitative analysis of denatonium benzoate by the isotopic dilution.

## Conclusions

In conclusion, we demonstrate a simple methodology of α-C deuterated denatonium cation synthesis. The reaction occurs at room temperature and is completed after 1 h incubation of denatonium benzoate in 1 % TEA/D_2_O solution. The introduced deuterons do not undergo back exchange under acidic and neutral conditions. Additionally, it was found that the isotopologues are characterized by practically the same retention time during chromatographic separation. The applicability of such deuterated standard in the quantitative analysis of denatonium benzoate was confirmed by the LC-MS analysis of several denatonium benzoate-containing samples. The obtained results indicate that the presented strategy is a new and simple solution for sensitive denatonium benzoate quantification. The presented methodology of synthesis of α-C deuterated isotopologues is not limited to denatonium cation and may be applied for deuteration of other betaine-containing compounds.

## Electronic supplementary material

ESM 1 (PDF 1008 kb)
