# Supplementary material for: Facile synthesis of deuterium-labeled denatonium cation and its application in the quantitative analysis of Bitrex by liquid chromatography-mass spectrometry
Source: Anal Bioanal Chem. 2015 Jun 16;407(21):6557–61. doi: 10.1007/s00216-015-8815-y (PMC4516867; doi:10.1007/s00216-015-8815-y)
Supplement: Supplementary file 1 — (PDF 1008 kb) [file 216_2015_8815_MOESM1_ESM.pdf]

Analytical and Bioanalytical Chemistry

Electronic Supplementary Material

Facile synthesis of deuterium labeled denatonium cation and its application in the quantitative analysis of Bitrex by liquid chromatography-mass spectrometry

Remigiusz Bąchor, Alicja Kluczyk, Piotr Stefanowicz, Zbigniew Szewczuk

Materials and Methods

Chemicals

Denatonium benzoate (≥ 98%), deuterium oxide (D_2O, 99.9% purity), *N*,*N*,*N*-triethylamine (TEA) and acetonitrile (LC/MS grade) were purchased from Sigma-Aldrich (Saint Louis, MO, USA).

Mass spectrometry

All ESI-MS experiments were performed on a micrOTOF-Q mass spectrometer (Bruker Daltonics, Bremen, Germany) equipped with standard ESI source. The instrument was operated in the positive-ion mode and calibrated with the Tunemix™ mixture (Agilent Technologies, Palo Alto, CA, USA). The mass accuracy was better than 5 ppm. Analyte solution was infused at a flow rate of 3 µl/min. The instrument parameters were as follows: scan range: 50-1600 *m/z*; drying gas: nitrogen; flow rate: 4.0 L/min, temperature: 200 ºC; potential between the spray needle and the orifice: 4.2 kV. For MS spectra analysis, a Bruker Compass DataAnalysis 4.0 software was used.

Isotopic exchange

HDX was initiated by dissolving 0.1 mg of the analyte in 198 µl of D_2O at room temperature (pD = 7.4). After 20 min, proton from the amide group was exchanged by deuterons, according to ESI-MS analysis. Then TEA (2 µL was added to the solution (pD = 12.3). To observe only the introduced α-C deuterons the sample was afterwards lyophilized and redissolved in the mixture of 200 µL of water/MeCN (1:1, v/v), incubated for 30 min and subjected to ESI-MS analysis.

In order to estimate pD of the analyzed alkaline solutions, pH was measured on a MP230 pH meter (Mettler-Toledo, Greifensee, Switzerland). pD was calculated according to the equation: pD = pH + 0.4 [1].

NMR analysis

NMR spectra were recorded on high-field spectrometer Bruker Avance 500 MHz. Complete ^1H NMR, ^{13}C NMR and COSY analysis was performed on each step of denatonium benzoate HDX. Samples were dissolved in $CDCl_3$.

Liquid Chromatography - Mass Spectrometry (LC-MS) analysis

The Agilent 1200 system, equipped with an Aeris Peptide XB-C18 column (50 mm × 2.1 mm) 3.6 μm bead diameter was used. The LC system was operated with mobile phase: solvent A: 0.1% formic acid in H_2O; solvent B: 0.1% formic acid in MeCN. The gradient conditions (B %) were form 0 to 70% B within 20 min. The flow rate was 0.1 mL/min and the injection volume 5 μL.

Preparation of alcoholic thinner samples for LC-MS analysis

The different volumes of alcoholic thinner (1300, 1000, 700, 500, 200, 50, 30, 20 µL) were evaporated under nitrogen stream and the residue was dissolved in H_2O (200 µL) before LC-MS analysis. To the 20 µL of such solution 2 µL of deuterated denatonium benzoate solution in H_2O (0.5 mg/mL) was added and diluted with H_2O to the total volume 145 µL. the prepared samples were analyzed by LC-MS.

Preparation of windscreen washer fluids samples for LC-MS analysis

The samples were prepared by the evaporating of 20 mL of each windscreen washer fluid. Then the sample was redissolved in H_2O (1 mL). To the 20 µL of such solution deuterated denatonium benzoate standard (2 µL, 0.5 mg/mL) was added. Then the sample was diluted to 145 µL with water and analyzed by LC-MS. Samples were evaporated both under nitrogen stream and by speedvac. The time required for evaporation was very short for low-volume samples (within minutes) whereas for larger amounts it took around 1 hour.

The computational analysis

The quantification of detected denatonium cations in the prepared samples was performed according to the algorithm described by Mirgorodskaya and co-workers [2]. Briefly, the relative concentrations of non-deuterated sample and isotopically labeled standard can be determined by comparing the isotopic distributions of “natural” and labeled compounds with isotopic pattern measured for analyzed sample containing known amount of deuterated standard. To reduce nonsystematical errors resulting from statistical fluctuations the data were analyzed by least squares method basing on abundancies of 6 isotopic peaks found in labeled and unlabeled compound. The comptutational analysis was peprformed using ORIGIN 8 program (OriginLab Corporation, Northampton, MA, USA)

NMR Analysis

In the ^{1}H NMR spectrum recorded for non-deuterated denatonium benzoate dissolved in $CDCl_3$ an intensive signal at 4.97 ppm, corresponding to the α-CH_2 group is present (Fig. 1S A). After the incubation in 1% TEA/D_2O solution the disappearance of the signal was observed. This clearly indicates that α-C hydrogens were replaced by deuterons (Fig. 1S B).

This result confirms that the specific deuteration of the α-C hydrogens takes place in denatonium cation.

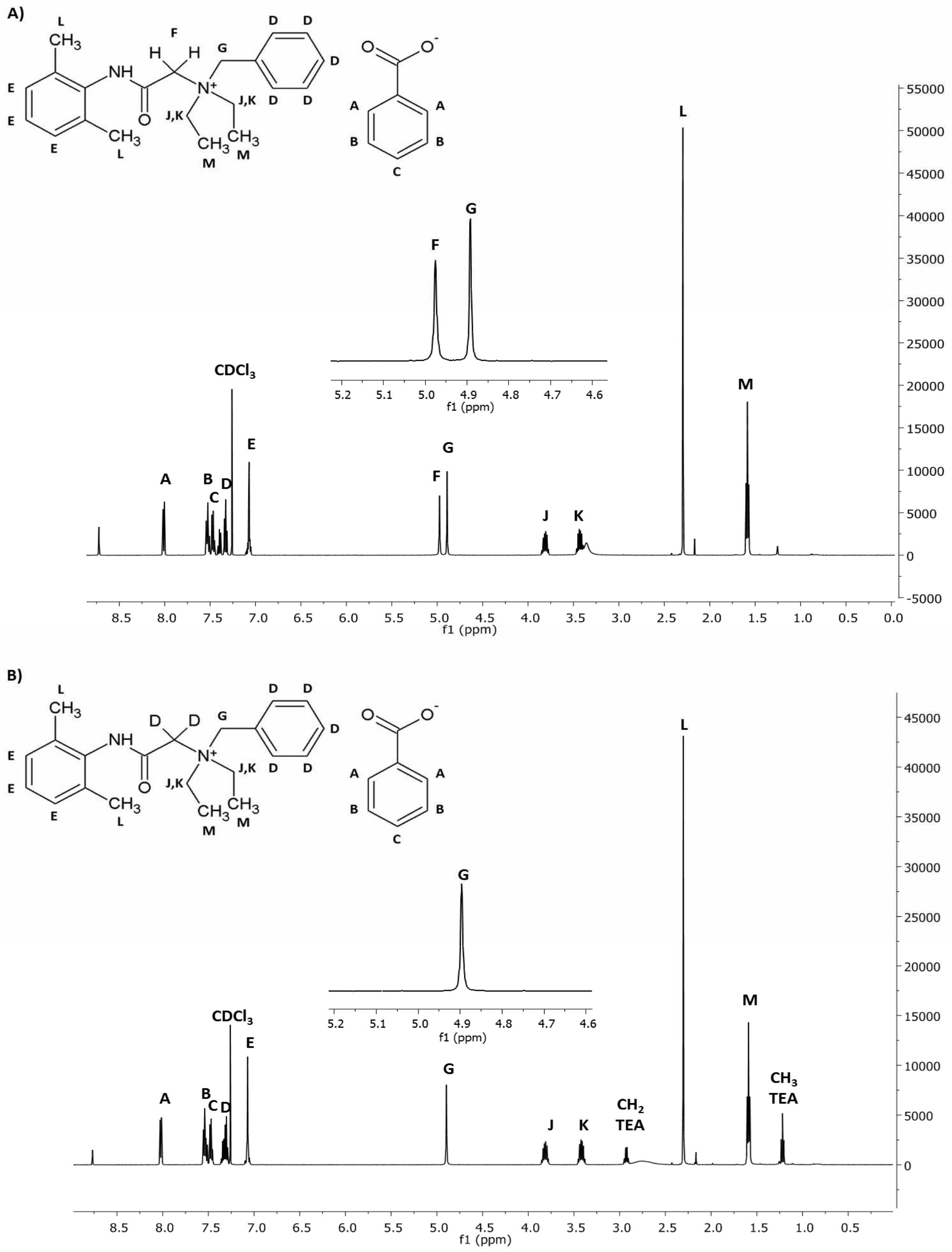

Fig. S1 The ^{1}H NMR spectra of denatonium benzoate before HDX at the α-C (A) and after incubation in 1% TEA/D_2O solution (B). The changes in resonance of the α-C hydrogens are presented in inserts. The disappearance of resonance signal at the 4.97 ppm confirms the exchange of the α-C hydrogens into deuterons (B)

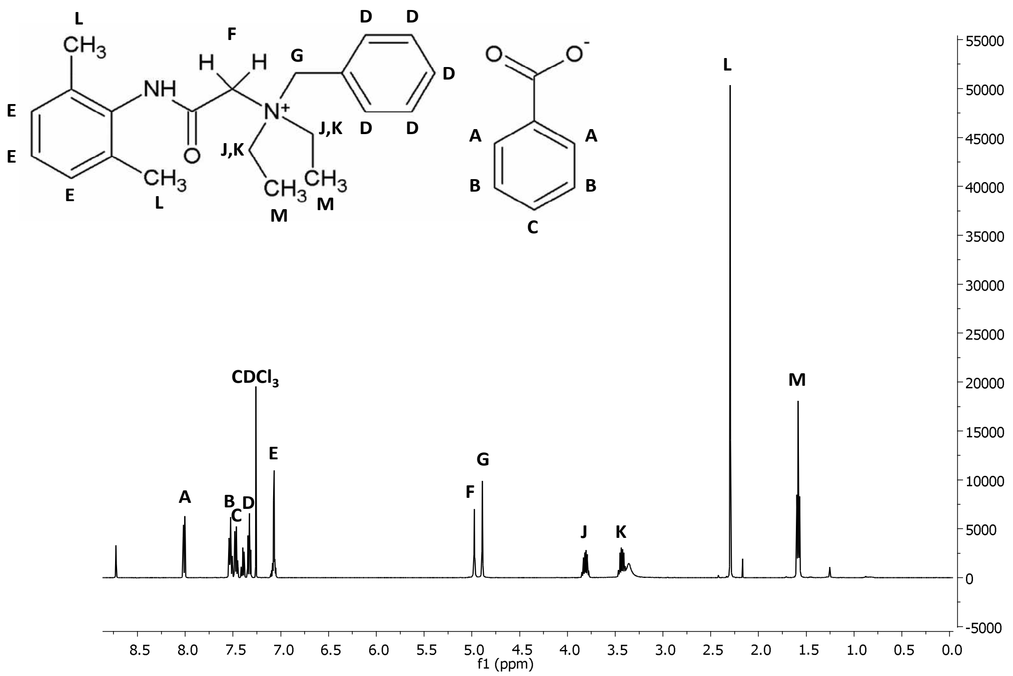

Fig. S2 The ^{1}H NMR spectrum of Bitrex dissolved in $CDCl_3$

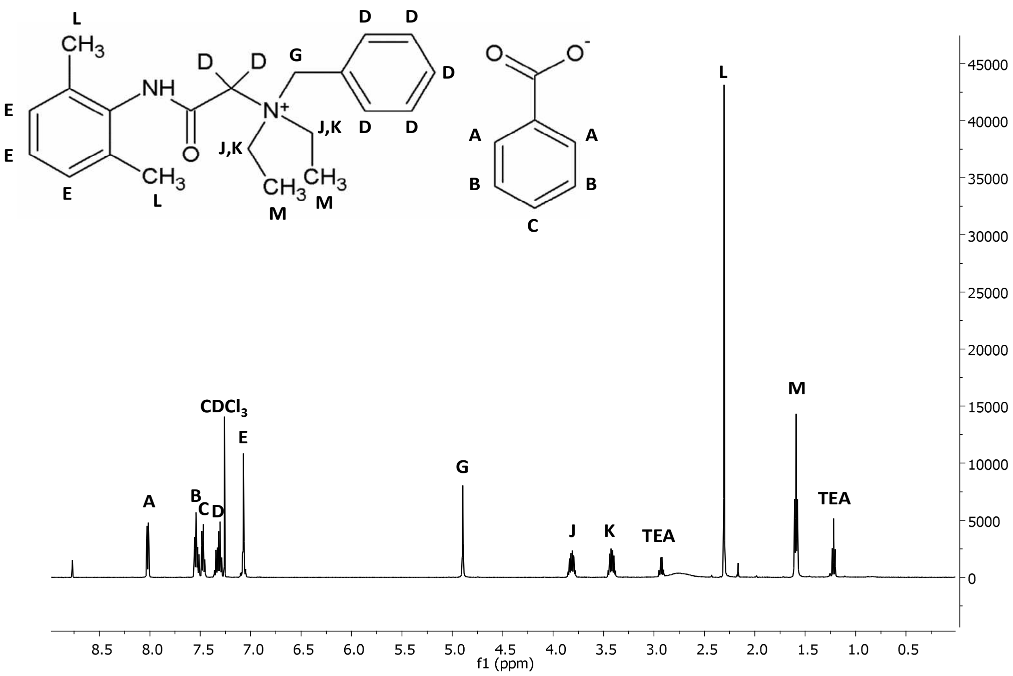

Fig. S3 The ^{1}H NMR spectrum of Bitrex after HDX in 1% TEA/D_2O solution. Sample was dissolved in $CDCl_3$

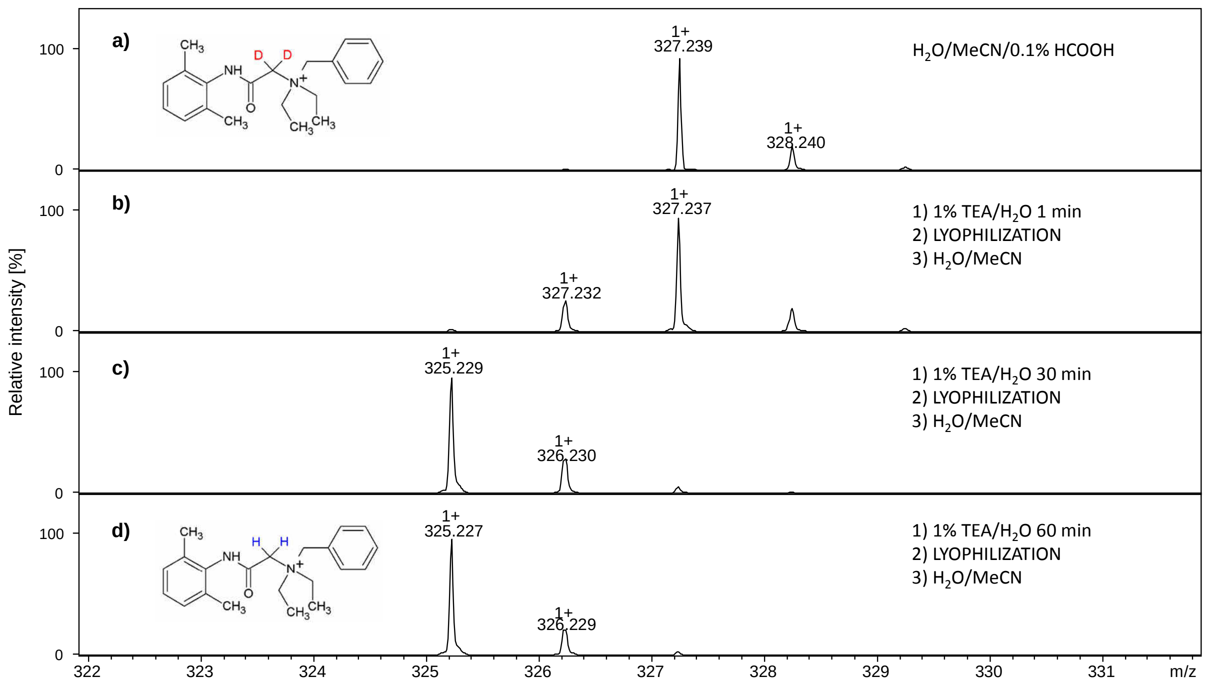

Fig. S4 ESI-MS spectra in positive ion mode of α-C deuterated Bitrex dissolved in: a) acetonitrile–water mixture; and after incubation in 1% TEA/H_2O for b) 1 min; c) 30 min; d) 60 min and. After back exchange samples were lyophilized and redissolved in acetonitrile–water

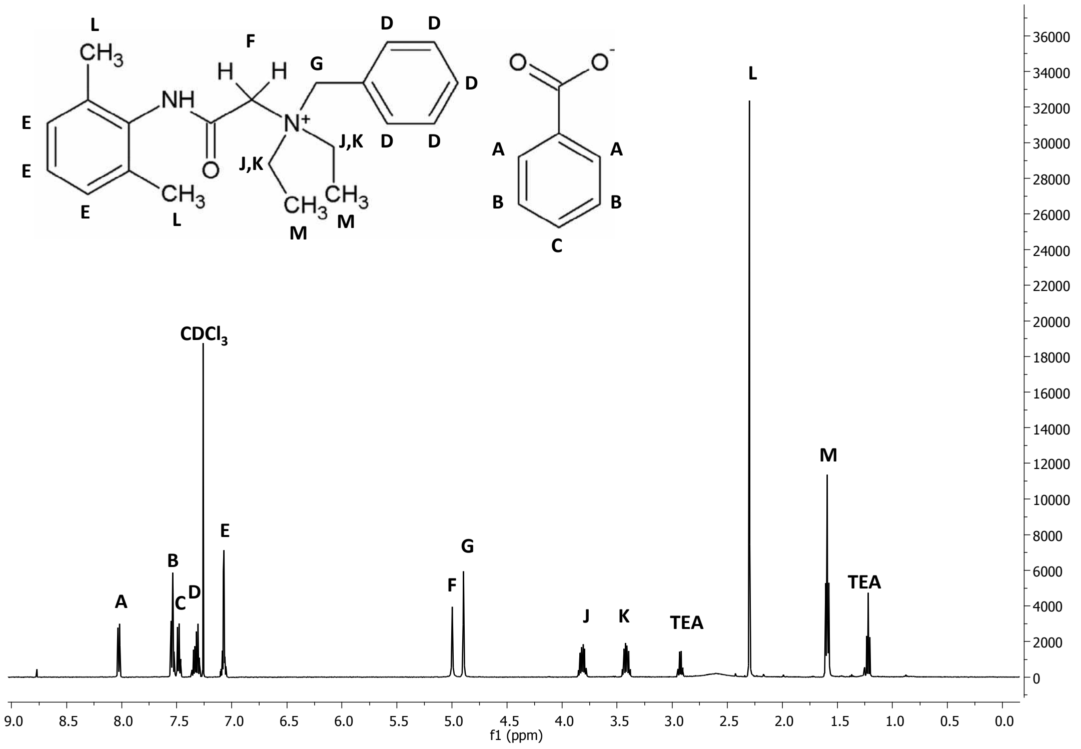

Fig. S5 The ^{1}H NMR spectrum of α-C deuterated Bitrex after back exchange in 1% TEA/H_2O solution. Sample was dissolved in $CDCl_3$

Quaternary ammonium compounds containing strong electron-withdrawing group attached to one of the methylene carbon bound to the quaternary nitrogen have been reported to undergo Stevens rearrangement in the presence of a strong base to give rearranged tertiary amines [3]. Additionally it was found by Ng and co-workers [4] that the presence of 2,6-xylycarbamoyl group close to the quaternary nitrogen atom facilitates the loss of a labile proton from the methylene group with subsequent migration of a benzylic group from the quaternary nitrogen during the GC-MS analysis of denatonium cation. To verify whether Stevens reaction occurs under proposed HDX conditions, the ^{13}C NMR and 2D NMR experiments were performed for denatonium benzoate sample after a six-hour incubation in 1% TEA/D_2O solution and compared with unexchanged sample (Figs. 6S - 9S). No differences have been found in ^{13}C and 2D NMR spectra of denatonium benzoate before and after HDX which clearly indicates that Stevens rearrangement does not occur under the reaction conditions.

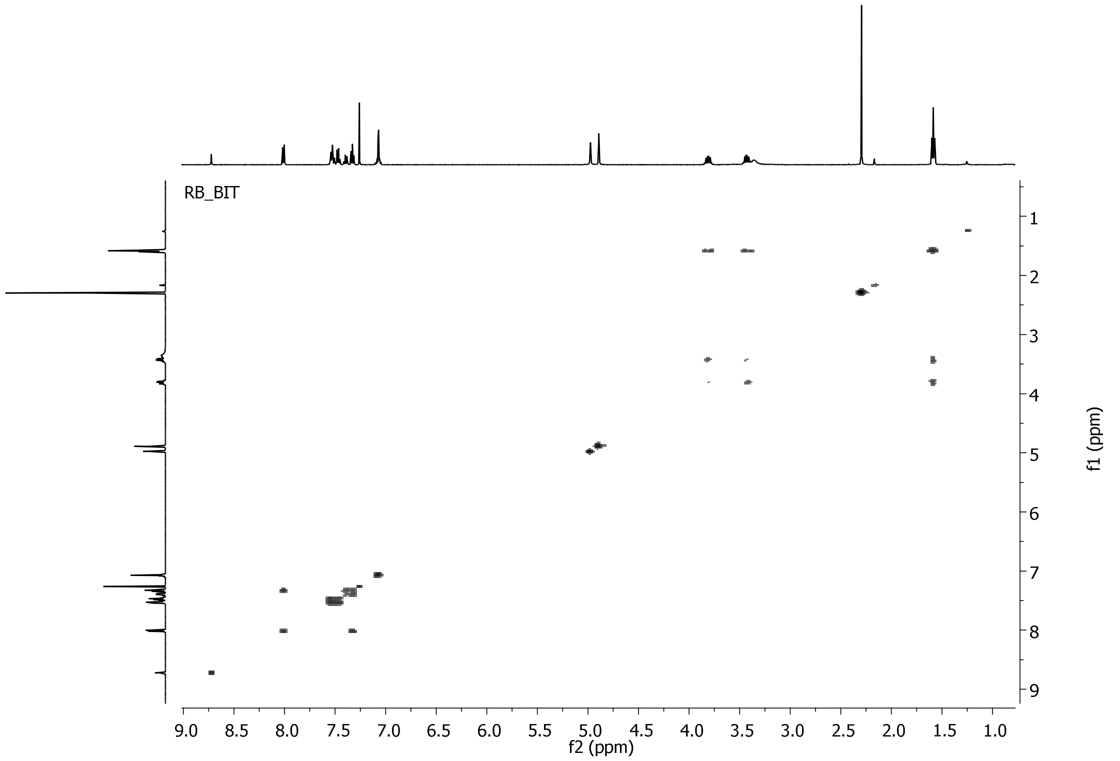

Fig. S6 The COSY spectrum of Bitrex. Sample was dissolved in $CDCl_3$

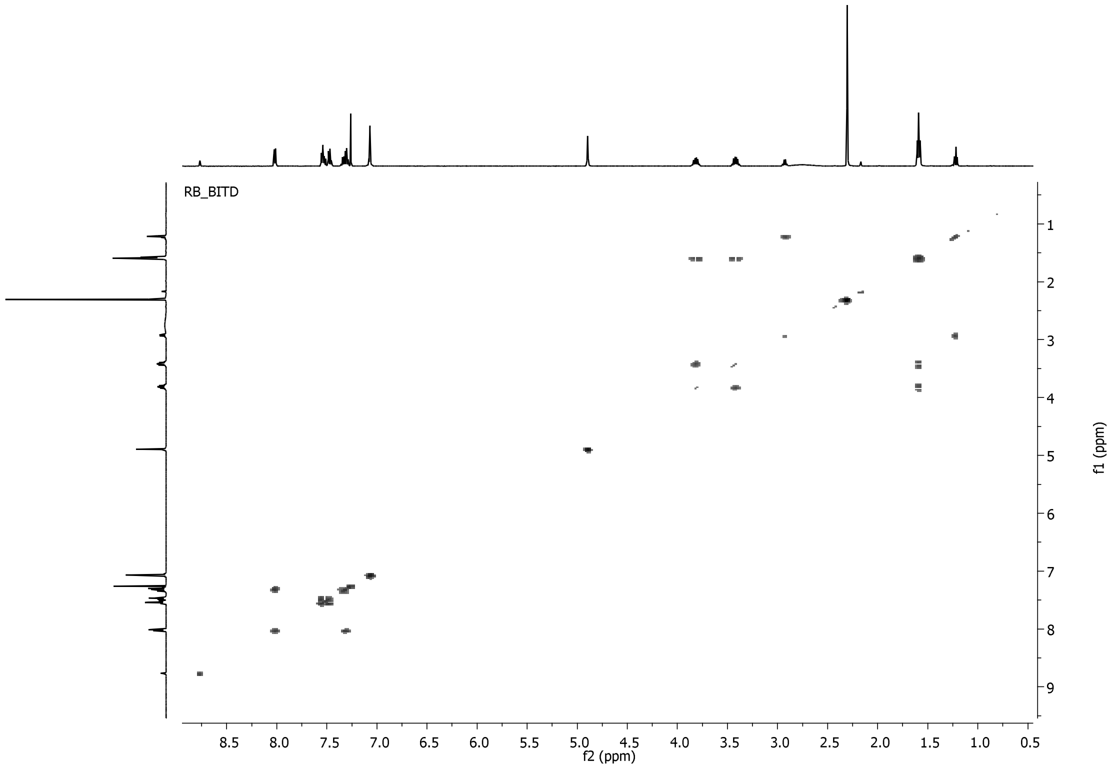

Fig. S7 The COSY spectrum of α-C deuterated Bitrex. Sample was dissolved in $CDCl_3$

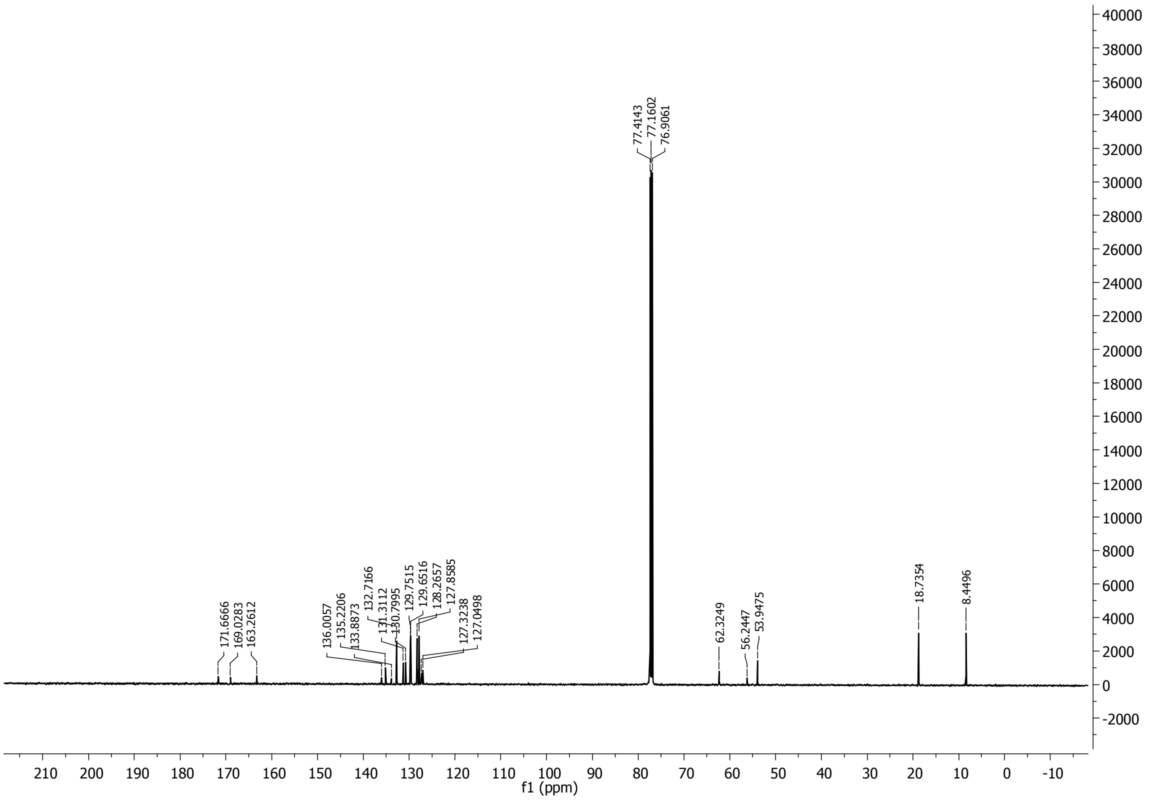

Fig. S8 The ^{13}C NMR spectrum of Bitrex. Sample was dissolved in $CDCl_3$

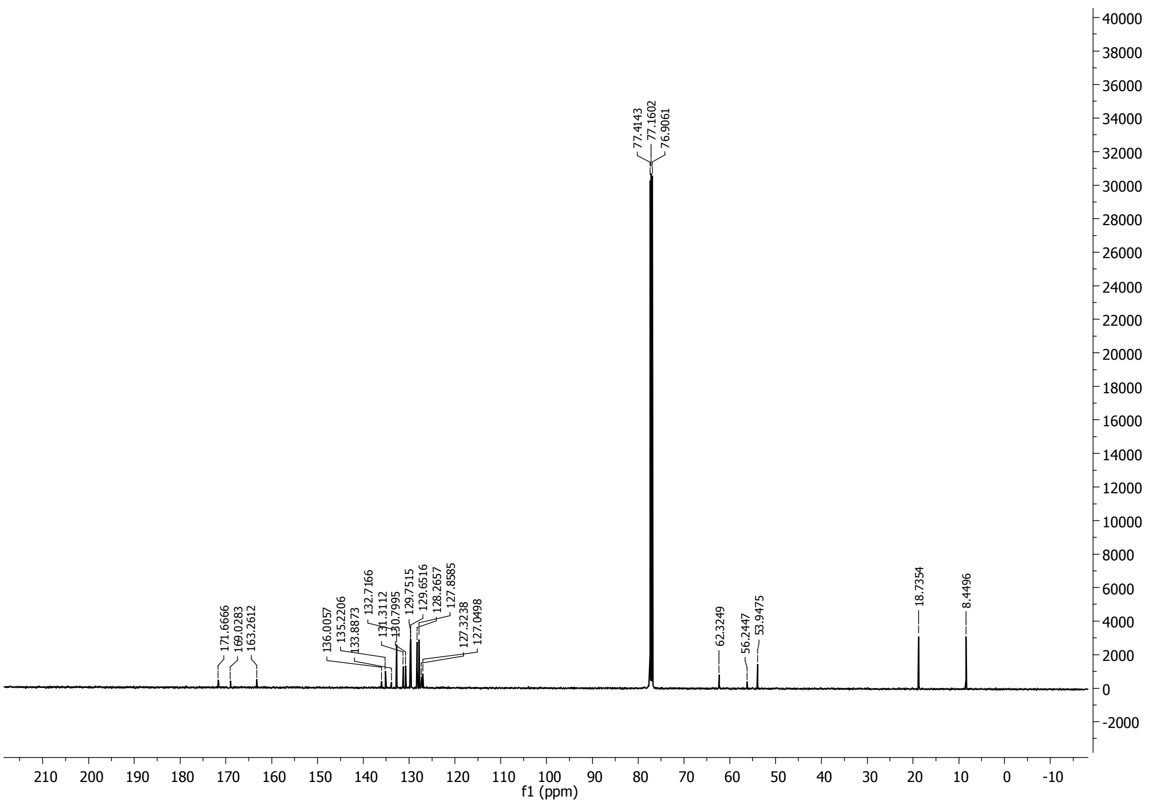

Fig. S9 The ^{13}C NMR spectrum of α-C deuterated Bitrex dissolved in $CDCl_3$

LC-MS analysis of spirit thinner samples.

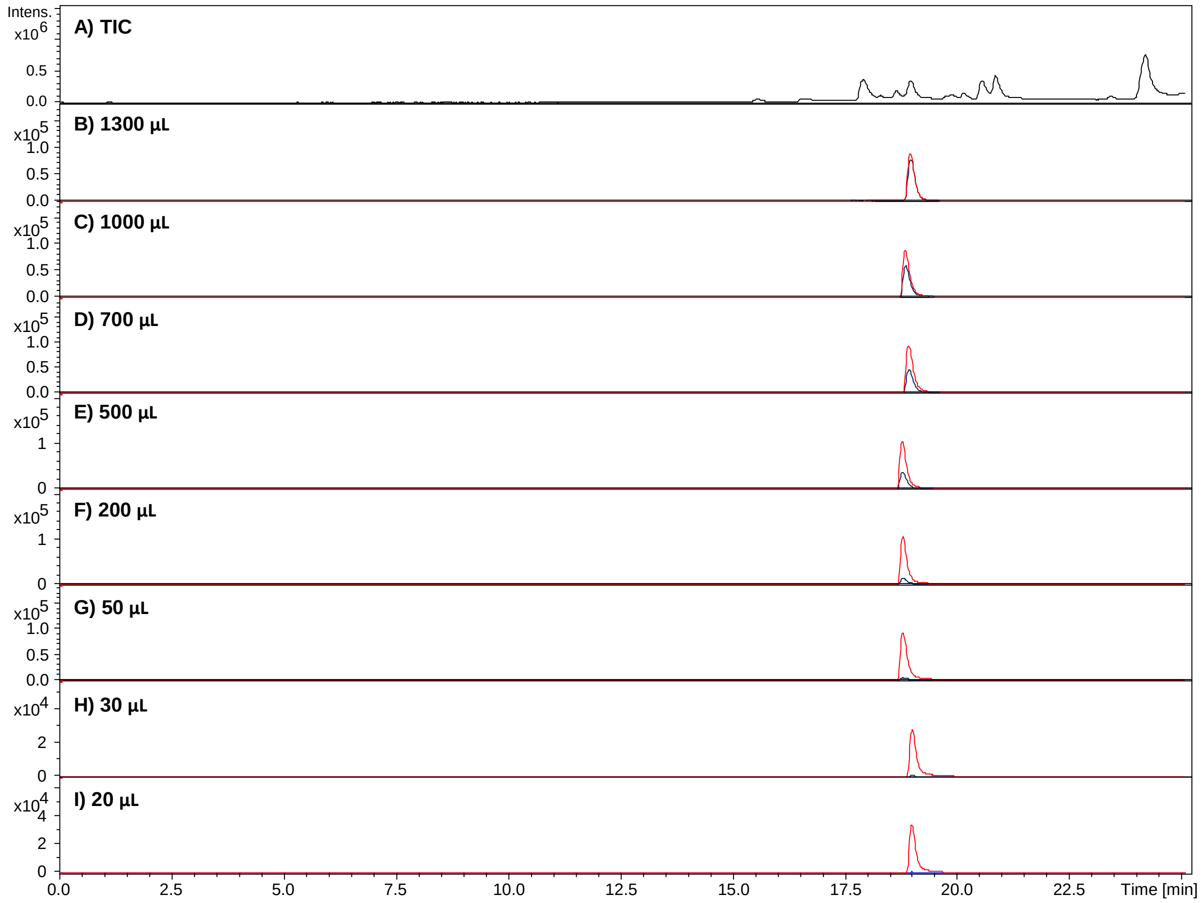

Fig. S10 Extracted ion chromatograms of denatonium cation (blue line) observed in the sample of spirit thinner and α-C deuterated denatonium cation (red line) as added standard. Total ion chromatogram is presented at the window A. The following volumes of spirit thinner were used during the experiments: B) 1300 µL; C) 1000 µL; D) 700 µL; E) 500 µL; F) 200 µL; G) 50 µL; H) 30 µL; I) 20 µL

The level of detected denatonium cations in used samples of spirit thinner was determined by the isotopic distribution observed on the ESI-MS according to the spectra of algorithm described by Mirgorodskaya and co-workers [2]. The obtained results are presented below.

Multiple Regression (2015-02-12 13:29:41)

Notes

Input Data

Masked Data - Values Excluded from Computations

Bad Data (missing values) -- Values that are invalid and thus not used in computations

Parameters

		Value	Standard Error
C	Intercept	-0,00255	0,00232
	A	0,48591	0,00616
	B	0,52937	0,00613

Statistics

	C
Number of Points	6
Degrees of Freedom	3
Residual Sum of Squares	4,84321E-5
Adj. R-Square	0,99953

Summary

	Intercept		A		B		Statistics
	Value	Standard Error	Value	Standard Error	Value	Standard Error	Adj. R-Square
C	-0,00255	0,00232	0,48591	0,00616	0,52937	0,00613	0,99953

ANOVA

		DF	Sum of Squares	Mean Square	F Value	Prob>F
C	Model	2	0,17271	0,08635	5349,01141	4,69401E-6
	Error	3	4,84321E-5	1,6144E-5		
	Total	5	0,17276			

Residual vs. Independent Plot

	ISOTOPE PATTERN			NORMALIZED VALUES				OBTAINED			CALCULATED ISOTOPE PATTERN		
m/z	H	D	LC MS	NH	ND	NLC		PARAMETERS			HA	DB	HA+DB
325,2	27228	379	68635	0,769522	0,004113	0,374858		A	0,48591		0,373918	0,0021772	0,3760957
326,2	6546	1149	16200	0,185004	0,012469	0,088478		B	0,52937		0,089895	0,0066006	0,096496
327,2	1387	71240	78020	0,0392	0,773087	0,426115		R^2	0,99953		0,019047	0,4092493	0,4282967
328,2	222	17006	17697	0,006274	0,184547	0,096654					0,003049	0,0976936	0,1007423
329,2	0	2198	2313	0	0,023852	0,012633					0	0,0126268	0,0126268
330,2	0	178	231	0	0,001932	0,001262					0	0,0010225	0,0010225
	35383	92150	183096										

Fig. S11 The results of computational analysis of LC-MS analysis of 1300 µL of spirit thinner with added deuterated Bitrex standard

Multiple Regression (2015-02-12 13:31:20)

Notes

Input Data

Masked Data - Values Excluded from Computations

Bad Data (missing values) -- Values that are invalid and thus not used in computations

Parameters

		Value	Standard Error
C	Intercept	-3,46049E-4	6,56625E-4
	A	0,41195	0,00174
	B	0,59012	0,00174

Statistics

	C
Number of Points	6
Degrees of Freedom	3
Residual Sum of Squares	3,88317E-6
Adj. R-Square	0,99996

Summary

	Intercept		A		B		Statistics
	Value	Standard Error	Value	Standard Error	Value	Standard Error	Adj. R-Square
C	-3,46049E-4	6,56625E-4	0,41195	0,00174	0,59012	0,00174	0,99996

ANOVA

		DF	Sum of Squares	Mean Square	F Value	Prob>F
C	Model	2	0,17742	0,08871	68534,6478	1,0239E-7
	Error	3	3,88317E-6	1,29439E-6		
	Total	5	0,17743			

Residual vs. Independent Plot

	ISOTOPE PATTERN			NORMALIZED VALUES				OBTAINED			CALCULATED ISOTOPE PATTERN		
m/z	H	D	LC MS	NH	ND	NLC		PARAMETERS			HA	DB	HA+DB
325,2	27228	379	39547	0,769522	0,004113	0,318729		A	0,41195		0,3170046	0,0024271	0,3194317
326,2	6546	1149	10510	0,185004	0,012469	0,084705		B	0,59012		0,0762124	0,0073581	0,0835705
327,2	1387	71240	58579	0,0392	0,773087	0,472118		R^2	0,99996		0,0161483	0,4562143	0,4723626
328,2	222	17006	13746	0,006274	0,184547	0,110786					0,0025847	0,1089048	0,1114895
329,2	0	2198	1560	0	0,023852	0,012573					0	0,0140758	0,0140758
330,2	0	178	135	0	0,001932	0,001088					0	0,0011399	0,0011399
	35383	92150	124077										

Fig. S12 The results of computational analysis of LC-MS analysis of 1000 µL of spirit thinner with added deuterated Bitrex standard

Multiple Regression (2015-02-12 13:29:41)

- *Notes*
- *Input Data*
- *Masked Data - Values Excluded from Computations*
- *Bad Data (missing values) -- Values that are invalid and thus not used in computations*
- *Parameters*

		Value	Standard Error
C	Intercept	-0,00255	0,00232
	A	0,48591	0,00616
	B	0,52937	0,00613

- *Statistics*

	C
Number of Points	6
Degrees of Freedom	3
Residual Sum of Squares	4,84321E-5
Adj. R-Square	0,99953

- *Summary*

	Intercept		A		B		Statistics
	Value	Standard Error	Value	Standard Error	Value	Standard Error	Adj. R-Square
C	-0,00255	0,00232	0,48591	0,00616	0,52937	0,00613	0,99953

- *ANOVA*

		DF	Sum of Squares	Mean Square	F Value	Prob>F
C	Model	2	0,17271	0,08635	5349,01141	4,69401E-6
	Error	3	4,84321E-5	1,6144E-5		
	Total	5	0,17276			

- *Residual vs. Independent Plot*

	ISOTOPE PATTERN			NORMALIZED VALUES				OBTAINED			CALCULATED ISOTOPE PATTERN		
m/z	H	D	LC MS	NH	ND	NLC		PARAMETERS			HA	DB	HA+DB
325,2	27228	379	36684	0,769522	0,004113	0,259533		A	0,33477		0,2576129	0,00279	0,2604029
326,2	6546	1149	8863	0,185004	0,012469	0,062704		B	0,67836		0,0619338	0,0084583	0,0703922
327,2	1387	71240	75690	0,0392	0,773087	0,535494		R^2	0,99966		0,0131229	0,5244315	0,5375544
328,2	222	17006	17610	0,006274	0,184547	0,124588					0,0021004	0,1251893	0,1272897
329,2	0	2198	2308	0	0,023852	0,016329					0	0,0161805	0,0161805
330,2	0	178	191	0	0,001932	0,001351					0	0,0013103	0,0013103
	35383	92150	141346										

Fig. S13 The results of computational analysis of LC-MS analysis of 700 μL of spirit thinner with added deuterated Bitrex standard

Multiple Regression (2015-02-12 13:35:21)

- Notes
- Input Data
- Masked Data - Values Excluded from Computations
- Bad Data (missing values) -- Values that are invalid and thus not used in computations

Parameters

		Value	Standard Error
C	Intercept	-0,00554	0,00341
	A	0,2745	0,00906
	B	0,75874	0,00901

Statistics

	C
Number of Points	6
Degrees of Freedom	3
Residual Sum of Squares	1,04592E-4
Adj. R-Square	0,9993

Summary

	Intercept		A		B		Statistics
	Value	Standard Error	Value	Standard Error	Value	Standard Error	Adj. R-Square
C	-0,00554	0,00341	0,2745	0,00906	0,75874	0,00901	0,9993

ANOVA

		DF	Sum of Squares	Mean Square	F Value	Prob>F
C	Model	2	0,24888	0,12444	3569,24168	8,60992E-6
	Error	3	1,04592E-4	3,48641E-5		
	Total	5	0,24898			

- Residual vs. Independent Plot

	ISOTOPE PATTERN			NORMALIZED VALUES				OBTAINED			CALCULATED ISOTOPE PATTERN		
m/z	H	D	LC MS	NH	ND	NLC		PARAMETERS			HA	DB	HA+DB
325,2	27228	379	28613	0,769522	0,004113	0,209847		A	0,2745		0,2112338	0,0031206	0,2143544
326,2	6546	1149	6860	0,185004	0,012469	0,050311		B	0,75874		0,0507836	0,0094606	0,0602442
327,2	1387	71240	80902	0,0392	0,773087	0,593332		R^2	0,99956		0,0107603	0,5865723	0,5973326
328,2	222	17006	17667	0,006274	0,184547	0,129569					0,0017223	0,1400231	0,1417454
329,2	0	2198	2151	0	0,023852	0,015775					0	0,0180978	0,0180978
330,2	0	178	159	0	0,001932	0,001166					0	0,0014656	0,0014656
	35383	92150	136352										

Fig. S14 The results of computational analysis of LC-MS analysis of 500 μL of spirit thinner with added deuterated Bitrex standard

Multiple Regression (2015-02-12 13:37:10)

Notes

Input Data

Masked Data - Values Excluded from Computations

Bad Data (missing values) -- Values that are invalid and thus not used in computations

Parameters

		Value	Standard Error
C	Intercept	-0,002	9,5436E-4
	A	0,10494	0,00254
	B	0,90709	0,00252

Statistics

	C
Number of Points	6
Degrees of Freedom	3
Residual Sum of Squares	8,20307E-6
Adj. R-Square	0,99996

Summary

	Intercept		A		B		Statistics
	Value	Standard Error	Value	Standard Error	Value	Standard Error	Adj. R-Square
C	-0,002	9,5436E-4	0,10494	0,00254	0,90709	0,00252	0,99996

ANOVA

		DF	Sum of Squares	Mean Square	F Value	Prob>F
C	Model	2	0,36365	0,18182	66495,90385	1,07135E-7
	Error	3	8,20307E-6	2,73436E-6		
	Total	5	0,36366			

Residual vs. Independent Plot

	ISOTOPE PATTERN			NORMALIZED VALUES				OBTAINED			CALCULATED ISOTOPE PATTERN		
m/z	H	D	LC MS	NH	ND	NLC		PARAMETERS			HA	DB	HA+DB
325,2	27228	379	9542	0,769522	0,004113	0,082638		A	0,10494		0,0807536	0,0037307	0,0844844
326,2	6546	1149	3240	0,185004	0,012469	0,02806		B	0,90709		0,0194143	0,0113103	0,0307247
327,2	1387	71240	81204	0,0392	0,773087	0,70326		R^2	0,99996		0,0041136	0,7012598	0,7053734
328,2	222	17006	19258	0,006274	0,184547	0,166782					0,0006584	0,1674007	0,1680591
329,2	0	2198	2041	0	0,023852	0,017676					0	0,0216363	0,0216363
330,2	0	178	183	0	0,001932	0,001585					0	0,0017522	0,0017522
	35383	92150	115468										

Fig. S15 The results of computational analysis of LC-MS analysis of 200 µL of spirit thinner with added deuterated Bitrex standard

Multiple Regression (2015-02-12 13:38:08)

Notes

Input Data

Masked Data - Values Excluded from Computations

Bad Data (missing values) -- Values that are invalid and thus not used in computations

Parameters

		Value	Standard Error
C	Intercept	-0,00369	0,00189
	A	0,0354	0,00503
	B	0,98675	0,00501

Statistics

	C
Number of Points	6
Degrees of Freedom	3
Residual Sum of Squares	3,23009E-5
Adj. R-Square	0,99988

Summary

	Intercept		A		B		Statistics
	Value	Standard Error	Value	Standard Error	Value	Standard Error	Adj. R-Square
C	-0,00369	0,00189	0,0354	0,00503	0,98675	0,00501	0,99988

ANOVA

		DF	Sum of Squares	Mean Square	F Value	Prob>F
C	Model	2	0,44505	0,22253	20667,53832	6,18239E-7
	Error	3	3,23009E-5	1,0767E-5		
	Total	5	0,44509			

Residual vs. Independent Plot

	ISOTOPE PATTERN			NORMALIZED VALUES				OBTAINED			CALCULATED ISOTOPE PATTERN		
m/z	H	D	LC MS	NH	ND	NLC		PARAMETERS			HA	DB	HA+DB
325,2	27228	379	1289	0,769522	0,004113	0,027368		A	0,0354		0,0272411	0,0040584	0,0312994
326,2	6546	1149	758	0,185004	0,012469	0,016094		B	0,98675		0,0065491	0,0123036	0,0188527
327,2	1387	71240	35874	0,0392	0,773087	0,761672		R^2	0,99988		0,0013877	0,762844	0,7642316
328,2	222	17006	8178	0,006274	0,184547	0,173634					0,0002221	0,1821017	0,1823238
329,2	0	2198	1000	0	0,023852	0,021232					0	0,0235364	0,0235364
330,2	0	178	0	0	0,001932	0					0	0,001906	0,001906
	35383	92150	47099										

Fig. S16 The results of computational analysis of LC-MS analysis of 50 µL of spirit thinner with added deuterated Bitrex standard

Multiple Regression (2015-02-12 13:40:34)

Notes

Input Data

Masked Data - Values Excluded from Computations

Bad Data (missing values) -- Values that are invalid and thus not used in computations

Parameters

		Value	Standard Error
C	Intercept	-0,00113	0,00124
	A	0,01836	0,0033
	B	0,98843	0,00329

Statistics

	C
Number of Points	6
Degrees of Freedom	3
Residual Sum of Squares	1,39309E-5
Adj. R-Square	0,99995

Summary

	Intercept		A		B		Statistics
	Value	Standard Error	Value	Standard Error	Value	Standard Error	Adj. R-Square
C	-0,00113	0,00124	0,01836	0,0033	0,98843	0,00329	0,99995

ANOVA

		DF	Sum of Squares	Mean Square	F Value	Prob>F
C	Model	2	0,45053	0,22527	48510,55414	1,71934E-7
	Error	3	1,39309E-5	4,64363E-6		
	Total	5	0,45054			

Residual vs. Independent Plot

	ISOTOPE PATTERN			NORMALIZED VALUES				OBTAINED			CALCULATED ISOTOPE PATTERN		
m/z	H	D	LC MS	NH	ND	NLC		PARAMETERS			HA	DB	HA+DB
325,2	27228	379	378	0,769522	0,004113	0,016384		A	0,01836		0,0141284	0,0040653	0,0181937
326,2	6546	1149	402	0,185004	0,012469	0,017424		B	0,98843		0,0033967	0,0123245	0,0157212
327,2	1387	71240	17616	0,0392	0,773087	0,763556		R^2	0,99995		0,0007197	0,7641427	0,7648624
328,2	222	17006	4204	0,006274	0,184547	0,18222					0,0001152	0,1824117	0,1825269
329,2	0	2198	471	0	0,023852	0,020415					0	0,0235764	0,0235764
330,2	0	178	0	0	0,001932	0					0	0,0019093	0,0019093
	35383	92150	23071										

Fig. S17 The results of computational analysis of LC-MS analysis of 30 μL of spirit thinner with added deuterated Bitrex standard

Multiple Regression (2015-02-12 13:40:34)

Notes

Input Data

Masked Data - Values Excluded from Computations

Bad Data (missing values) -- Values that are invalid and thus not used in computations

Parameters

		Value	Standard Error
C	Intercept	-0,00113	0,00124
	A	0,01836	0,0033
	B	0,98843	0,00329

Statistics

	C
Number of Points	6
Degrees of Freedom	3
Residual Sum of Squares	1,39309E-5
Adj. R-Square	0,99995

Summary

	Intercept		A		B		Statistics
	Value	Standard Error	Value	Standard Error	Value	Standard Error	Adj. R-Square
C	-0,00113	0,00124	0,01836	0,0033	0,98843	0,00329	0,99995

ANOVA

		DF	Sum of Squares	Mean Square	F Value	Prob>F
C	Model	2	0,45053	0,22527	48510,55414	1,71934E-7
	Error	3	1,39309E-5	4,64363E-6		
	Total	5	0,45054			

Residual vs. Independent Plot

	ISOTOPE PATTERN			NORMALIZED VALUES				OBTAINED			CALCULATED ISOTOPE PATTERN		
m/z	H	D	LC MS	NH	ND	NLC		PARAMETERS			HA	DB	HA+DB
325,2	27228	379	363	0,769522	0,004113	0,010609		A	0,01315		0,010119215	0,004119	0,0142387
326,2	6546	1149	591	0,185004	0,012469	0,017273		B	1,0016		0,002432804	0,012489	0,0149216
327,2	1387	71240	26461	0,0392	0,773087	0,773352		R^2	0,99984		0,000515475	0,774324	0,7748398
328,2	222	17006	6098	0,006274	0,184547	0,178221					8,25057E-05	0,184842	0,1849247
329,2	0	2198	703	0	0,023852	0,020546					0	0,023891	0,0238906
330,2	0	178	0	0	0,001932	0					0	0,001935	0,0019347
	35383	92150	34216										

Fig. S18 The results of computational analysis of LC-MS analysis of 20 µL of spirit thinner with added deuterated Bitrex standard

To match the analyte concentration in the sample we prepared the solution of denatonium benzoate in the ethanol with concentration 0.8 mg/mL. Then sample was evaporated under nitrogen stream and dissolved in 1mL of water. To the 20, 30 and 40μL of this solution 20 μL of the stock solution of deuterated denatonium benzoate standard (concentration 1 mg/mL) were added followed by the addition of 200 μL of water. The quantification of denatonium benzoate was performed by LC-MS analysis. The obtained results (presented below) are in agreement with the theoretical amount of denatonium benzoate used for the denaturation of ethanol sample. The obtained results are presented below.

Multiple Regression (2015-05-11 18:35:11)

Notes

Input Data

Masked Data - Values Excluded from Computations

Bad Data (missing values) -- Values that are invalid and thus not used in computations

Parameters

		Value	Standard Error
C	Intercept	-1,18456E-4	0,00595
	A	0,37327	0,01611
	B	0,62744	0,01563

Statistics

	C
Number of Points	6
Degrees of Freedom	3
Residual Sum of Squares	3,13154E-4
Adj. R-Square	0,99716

Summary

	Intercept		A		B		Statistics
	Value	Standard Error	Value	Standard Error	Value	Standard Error	Adj. R-Square
C	-1,18456E-4	0,00595	0,37327	0,01611	0,62744	0,01563	0,99716

ANOVA

		DF	Sum of Squares	Mean Square	F Value	Prob>F
C	Model	2	0,18353	0,09176	879,08622	7,03038E-5
	Error	3	3,13154E-4	1,04385E-4		
	Total	5	0,18384			

Residual vs. Independent Plot

	ISOTOPE PATTERN			NORMALIZED VALUES				OBTAINED			CALCULATED ISOTOPE PATTERN		
m/z	H	D	LC MS	NH	ND	NLC		PARAMETERS			HA	DB	HA+DB
325,2	27228	379	67518	0,755444	0,004113	0,285892		A	0,37327		0,2819844	0,0025806	0,284565
326,2	6546	1149	16557	0,181619	0,012469	0,070107		B	0,62744		0,0677931	0,0078234	0,075617
327,2	1387	71240	117106	0,038482	0,773087	0,495863		R^2	0,99716		0,0143643	0,4850659	0,49943
328,2	222	17006	31499	0,006159	0,184547	0,133377					0,0022991	0,1157921	0,118091
329,2	329,2	2198	3220	0,009134	0,023852	0,013634					0,0034093	0,014966	0,018375
330,2	330,2	178	266	0,009161	0,001932	0,001126					0,0034197	0,001212	0,004632
	36042,4	92150	236166										

Fig. S19 The results of computational analysis of LC-MS analysis of 20 μL of denaturated spirit thinner with added deuterated denatonium benzoate standard

Multiple Regression (2015-05-11 18:42:26)

Notes

Input Data

Masked Data - Values Excluded from Computations

Bad Data (missing values) -- Values that are invalid and thus not used in computations

Parameters

		Value	Standard Error
C	Intercept	-2,46745E-4	0,00364
	A	0,46548	0,00984
	B	0,536	0,00955

Statistics

	C
Number of Points	6
Degrees of Freedom	3
Residual Sum of Squares	1,16842E-4
Adj. R-Square	0,99881

Summary

	Intercept		A		B		Statistics
	Value	Standard Error	Value	Standard Error	Value	Standard Error	Adj. R-Square
C	-2,46745E-4	0,00364	0,46548	0,00984	0,536	0,00955	0,99881

ANOVA

		DF	Sum of Squares	Mean Square	F Value	Prob>F
C	Model	2	0,16392	0,08196	2104,40019	1,90099E-5
	Error	3	1,16842E-4	3,89473E-5		
	Total	5	0,16404			

Residual vs. Independent Plot

	ISOTOPE PATTERN			NORMALIZED VALUES				OBTAINED			CALCULATED ISOTOPE PATTERN		
m/z	H	D	LC MS	NH	ND	NLC		PARAMETERS			HA	DB	HA+DB
325,2	27228	379	130131	0,755444	0,004113	0,353386		A	0,46548		0,3516439	0,0022045	0,353848
326,2	6546	1149	34024	0,181619	0,012469	0,092396		B	0,536		0,0845402	0,0066833	0,091223
327,2	1387	71240	158361	0,038482	0,773087	0,430048		R^2	0,99881		0,0179128	0,4143748	0,432288
328,2	222	17006	40648	0,006159	0,184547	0,110385					0,0028671	0,0989172	0,101784
329,2	329,2	2198	4629	0,009134	0,023852	0,012571					0,0042515	0,0127849	0,017036
330,2	330,2	178	447	0,009161	0,001932	0,001214					0,0042645	0,0010354	0,0053
	36042,4	92150	368240										

Fig. S20 The results of computational analysis of LC-MS analysis of 30 µL of denaturated spirit thinner with added deuterated denatonium benzoate standard

Multiple Regression (2015-05-11 18:47:12)

- *Notes*
- *Input Data*
- *Masked Data - Values Excluded from Computations*
- *Bad Data (missing values) -- Values that are invalid and thus not used in computations*

Parameters

		Value	Standard Error
C	Intercept	0,00255	0,00575
	A	0,50052	0,01557
	B	0,48419	0,01511

Statistics

	C
Number of Points	6
Degrees of Freedom	3
Residual Sum of Squares	2,92538E-4
Adj. R-Square	0,99688

Summary

	Intercept		A		B		Statistics
	Value	Standard Error	Value	Standard Error	Value	Standard Error	Adj. R-Square
C	0,00255	0,00575	0,50052	0,01557	0,48419	0,01511	0,99688

ANOVA

		DF	Sum of Squares	Mean Square	F Value	Prob>F
C	Model	2	0,15601	0,07801	799,95204	8,09693E-5
	Error	3	2,92538E-4	9,75128E-5		
	Total	5	0,1563			

- *Residual vs. Independent Plot*

	ISOTOPE PATTERN			NORMALIZED VALUES				OBTAINED			CALCULATED ISOTOPE PATTERN		
m/z	H	D	LC MS	NH	ND	NLC		PARAMETERS			HA	DB	HA+DB
325,2	27228	379	154172	0,755444	0,004113	0,381099		A	0,50052		0,378115	0,001991	0,380106
326,2	6546	1149	43223	0,181619	0,012469	0,106843		B	0,48419		0,090904	0,006037	0,0969414
327,2	1387	71240	159228	0,038482	0,773087	0,393597		R^2	0,99688		0,019261	0,374321	0,3935824
328,2	222	17006	42952	0,006159	0,184547	0,106173					0,003083	0,089356	0,0924387
329,2	329,2	2198	4546	0,009134	0,023852	0,011237					0,004572	0,011549	0,0161207
330,2	330,2	178	425	0,009161	0,001932	0,001051					0,004585	0,000935	0,0055208
	36042,4	92150	404546										

Fig. S21 The results of computational analysis of LC-MS analysis of 40 µL of denaturated spirit thinner with added deuterated denatonium benzoate standard

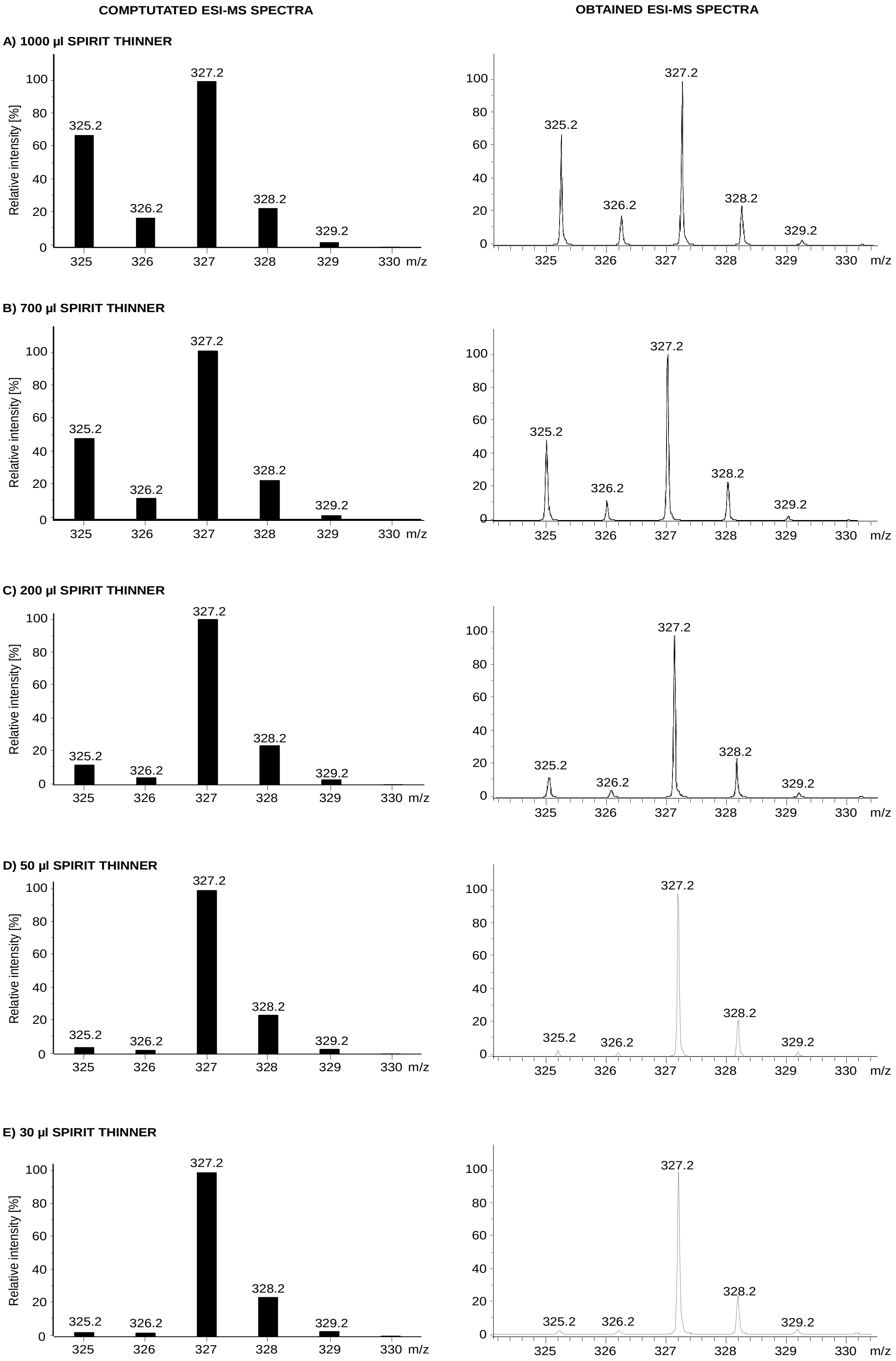

Fig. S22 Calculated and obtained isotopic patterns for LC-MS analysis of following volumes of spirit thinner: A) 1000µL; B) 700 µL; C) 200 µL;D) 50 µL; E) 30 µL

The experimental isotopic patterns for analyzed sample were compared with those obtained according to the computational procedure. The results obtained for the analysis of 1300, 500 and 20 µL of alcoholic thinner are presented below (Fig. 4A-C). Other spectra are presented in supplementary data (Fig. 23S). The same results were obtained when deuterated denatonium benzoate standard for quantitative analysis was directly added to the analyze sample (experiment not shown in experimental section).

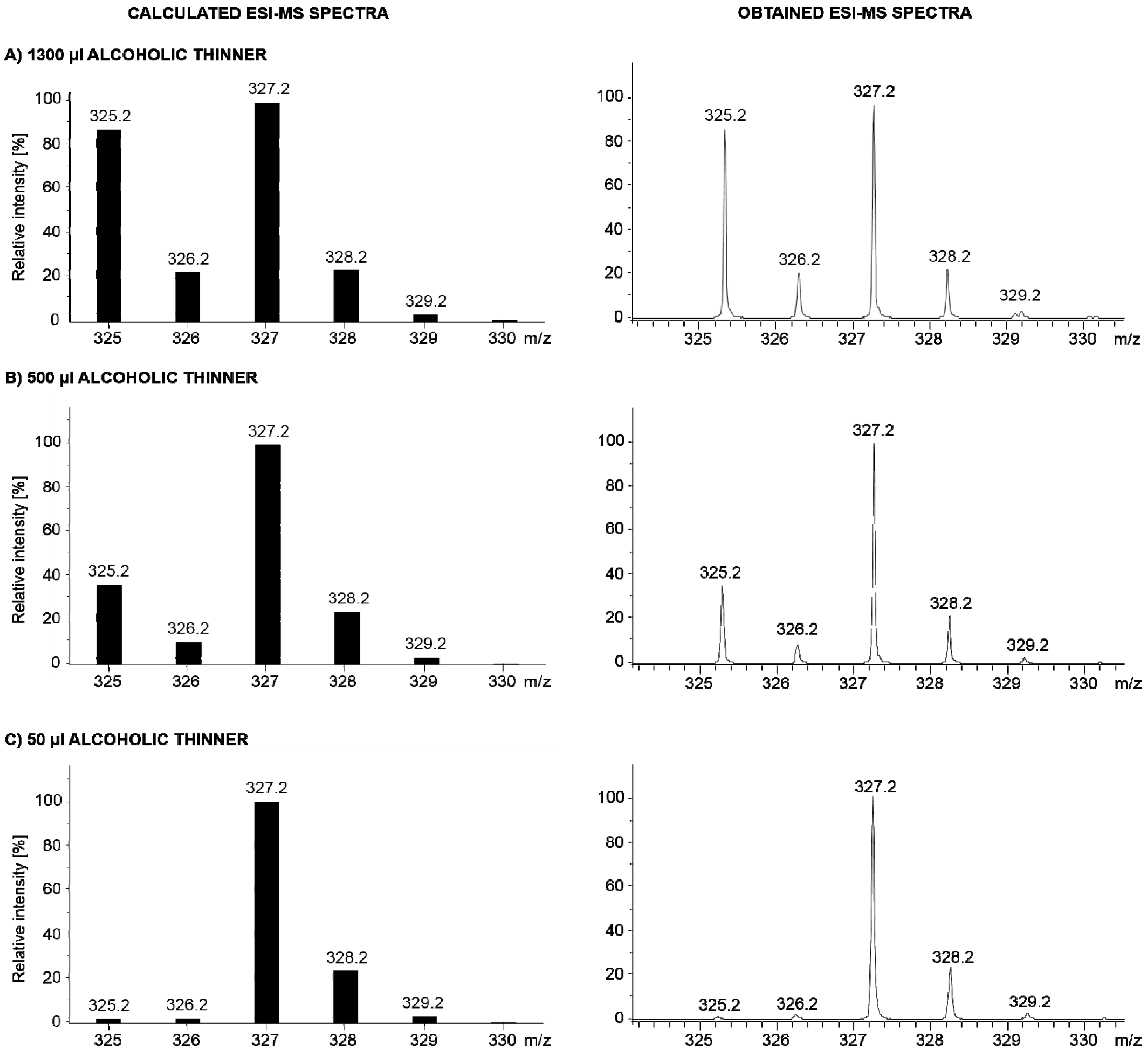

Fig. S23 Calculated and experimental isotopic distributions obtained after LC-MS analysis of denatonium cation in alcoholic thinner samples A) 1300 µL; B) 500 µL and C) 50 µL, containing deuterated denatonium benzoate standard

To the samples of 200, 500, 700 and 1000µL the deuterated isotopologue of denatonium benzoate was added (20 µl of the standard solution containing 1mg/mL of denatonium benzoate) and the samples were evaporated under nitrogen stream. The used volumes of deuterated standard (20 µL) do not lead to the pipetting volume errors like in the case of addition of 2 µL. The obtained results were reproducible and comparable with those obtained by us previously. Such analysis revealed the applicability of the proposed methodology in the quantitative analysis of denatonium benzoate.

Multiple Regression (2015-05-12 08:50:19)

Notes

Input Data

Masked Data - Values Excluded from Computations

Bad Data (missing values) -- Values that are invalid and thus not used in computations

Parameters

		Value	Standard Error
C	Intercept	-0,00149	0,00173
	A	0,32875	0,00469
	B	0,68022	0,00456

Statistics

	C
Number of Points	6
Degrees of Freedom	3
Residual Sum of Squares	2,66032E-5
Adj. R-Square	0,99978

Summary

	Intercept		A		B		Statistics
	Value	Standard Error	Value	Standard Error	Value	Standard Error	Adj. R-Square
C	-0,00149	0,00173	0,32875	0,00469	0,68022	0,00456	0,99978

ANOVA

		DF	Sum of Squares	Mean Square	F Value	Prob>F
C	Model	2	0,20472	0,10236	11542,96086	1,48107E-6
	Error	3	2,66032E-5	8,86772E-6		
	Total	5	0,20475			

Residual vs. Independent Plot

	ISOTOPE PATTERN			NORMALIZED VALUES				OBTAINED			CALCULATED ISOTOPE PATTERN		
m/z	H	D	LC MS	NH	ND	NLC		PARAMETERS			HA	DB	HA+DB
325,2	27228	379	109297	0,755444	0,004113	0,249688		A	0,32875		0,2483521	0,002798	0,2511497
326,2	6546	1149	29237	0,181619	0,012469	0,066792		B	0,68022		0,0597074	0,008482	0,0681889
327,2	1387	71240	234646	0,038482	0,773087	0,536046		R^2	0,99978		0,0126511	0,525869	0,5385206
328,2	222	17006	57100	0,006159	0,184547	0,130444					0,0020249	0,125533	0,1275574
329,2	329,2	2198	6853	0,009134	0,023852	0,015656					0,0030027	0,016225	0,0192276
330,2	330,2	178	602	0,009161	0,001932	0,001375					0,0030118	0,001314	0,0043258
	36042,4	92150	437735										

Fig. S24 The results of computational analysis of LC-MS analysis of 1000 µL of spirit thinner with added deuterated denatonium benzoate standard

Multiple Regression (2015-05-12 08:58:39)

- Notes
- Input Data
- Masked Data - Values Excluded from Computations
- Bad Data (missing values) -- Values that are invalid and thus not used in computations
- Parameters

		Value	Standard Error
C	Intercept	0,00413	0,01009
	A	0,26621	0,02731
	B	0,709	0,0265

- Statistics

	C
Number of Points	6
Degrees of Freedom	3
Residual Sum of Squares	9,00133E-4
Adj. R-Square	0,99309

- Summary

	Intercept		A		B		Statistics
	Value	Standard Error	Value	Standard Error	Value	Standard Error	Adj. R-Square
C	0,00413	0,01009	0,26621	0,02731	0,709	0,0265	0,99309

- ANOVA

		DF	Sum of Squares	Mean Square	F Value	Prob>F
C	Model	2	0,2161	0,10805	360,11807	2,67154E-4
	Error	3	9,00133E-4	3,00044E-4		
	Total	5	0,217			

- Residual vs. Independent Plot

	ISOTOPE PATTERN			NORMALIZED VALUES				OBTAINED			CALCULATED ISOTOPE PATTERN		
m/z	H	D	LC MS	NH	ND	NLC		PARAMETERS			HA	DB	HA+DB
325,2	27228	379	88901	0,755444	0,004113	0,210682		A	0,26621		0,201107	0,002916	0,204023
326,2	6546	1149	21867	0,181619	0,012469	0,051822		B	0,709		0,048349	0,00884	0,057189
327,2	1387	71240	234879	0,038482	0,773087	0,556629		R^2	0,99309		0,010244	0,548119	0,558363
328,2	222	17006	68660	0,006159	0,184547	0,162714					0,00164	0,130844	0,132483
329,2	329,2	2198	7107	0,009134	0,023852	0,016843					0,002431	0,016911	0,019343
330,2	330,2	178	553	0,009161	0,001932	0,001311					0,002439	0,00137	0,003808
	36042,4	92150	421967										

Fig. S25 The results of computational analysis of LC-MS analysis of 700 µL of spirit thinner with added deuterated denatonium benzoate standard

Multiple Regression (2015-05-12 09:04:01)

Notes

Input Data

Masked Data - Values Excluded from Computations

Bad Data (missing values) -- Values that are invalid and thus not used in computations

Parameters

		Value	Standard Error
C	Intercept	-0,00274	0,00341
	A	0,1762	0,00923
	B	0,84021	0,00896

Statistics

	C
Number of Points	6
Degrees of Freedom	3
Residual Sum of Squares	1,02825E-4
Adj. R-Square	0,99944

Summary

	Intercept		A		B		Statistics
	Value	Standard Error	Value	Standard Error	Value	Standard Error	Adj. R-Square
C	-0,00274	0,00341	0,1762	0,00923	0,84021	0,00896	0,99944

ANOVA

		DF	Sum of Squares	Mean Square	F Value	Prob>F
C	Model	2	0,30391	0,15195	4433,39939	6,2203E-6
	Error	3	1,02825E-4	3,42749E-5		
	Total	5	0,30401			

Residual vs. Independent Plot

	ISOTOPE PATTERN			NORMALIZED VALUES				OBTAINED			CALCULATED ISOTOPE PATTERN		
m/z	H	D	LC MS	NH	ND	NLC		PARAMETERS			HA	DB	HA+DB
325,2	27228	379	50432	0,755444	0,004113	0,13538		A	0,1762		0,133109	0,003456	0,136565
326,2	6546	1149	12460	0,181619	0,012469	0,033448		B	0,84021		0,032001	0,010476	0,042478
327,2	1387	71240	242880	0,038482	0,773087	0,651988		R^2	0,99944		0,006781	0,649556	0,656336
328,2	222	17006	59892	0,006159	0,184547	0,160774					0,001085	0,155058	0,156143
329,2	329,2	2198	6371	0,009134	0,023852	0,017102					0,001609	0,020041	0,02165
330,2	330,2	178	487	0,009161	0,001932	0,001307					0,001614	0,001623	0,003237
	36042,4	92150	372522										

Fig.S26 The results of computational analysis of LC-MS analysis of 500 µL of spirit thinner with added deuterated denatonium benzoate standard

Multiple Regression (2015-05-12 09:07:53)

Notes

Input Data

Masked Data - Values Excluded from Computations

Bad Data (missing values) -- Values that are invalid and thus not used in computations

Parameters

		Value	Standard Error
C	Intercept	0,00317	0,00904
	A	0,07642	0,02448
	B	0,90455	0,02375

Statistics

	C
Number of Points	6
Degrees of Freedom	3
Residual Sum of Squares	7,23118E-4
Adj. R-Square	0,99671

Summary

	Intercept		A		B		Statistics
	Value	Standard Error	Value	Standard Error	Value	Standard Error	Adj. R-Square
C	0,00317	0,00904	0,07642	0,02448	0,90455	0,02375	0,99671

ANOVA

		DF	Sum of Squares	Mean Square	F Value	Prob>F
C	Model	2	0,36569	0,18284	758,56443	8,76721E-5
	Error	3	7,23118E-4	2,41039E-4		
	Total	5	0,36641			

Residual vs. Independent Plot

	ISOTOPE PATTERN			NORMALIZED VALUES				OBTAINED			CALCULATED ISOTOPE PATTERN		
m/z	H	D	LC MS	NH	ND	NLC		PARAMETERS			HA	DB	HA+DB
325,2	27228	379	50348	0,755444	0,004113	0,067619		A	0,07642		0,057731	0,0037203	0,0614513
326,2	6546	1149	12376	0,181619	0,012469	0,016621		B	0,90455		0,013879	0,0112787	0,025158
327,2	1387	71240	521464	0,038482	0,773087	0,700342		R^2	0,99671		0,002941	0,6992962	0,702237
328,2	222	17006	143852	0,006159	0,184547	0,193198					0,000471	0,1669319	0,1674026
329,2	329,2	2198	15295	0,009134	0,023852	0,020542					0,000698	0,0215757	0,0222737
330,2	330,2	178	1250	0,009161	0,001932	0,001679					0,0007	0,0017473	0,0024474
	36042,4	92150	744585										

Fig. S27 The results of computational analysis of LC-MS analysis of 200 µL of spirit thinner with added deuterated denatonium benzoate standard

The LC-MS analysis of commercially available winter/summer windscreen washer fluids.

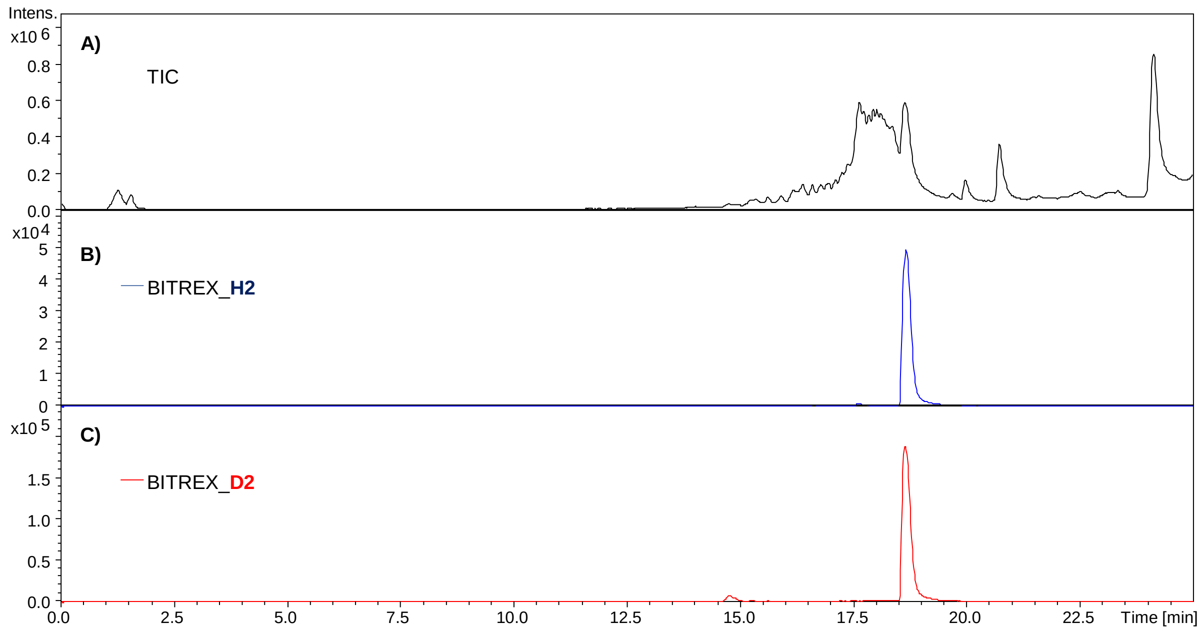

Fig. S28 The LC-MS analysis of commercially available winter windscreen washer fluid. Total ion chromatogram (A) and extracted ion chromatograms of detected denatonium cation (B) and added deuterated standard (C)

Multiple Regression (2015-05-11 10:57:33)

Notes

Input Data

Masked Data - Values Excluded from Computations

Bad Data (missing values) -- Values that are invalid and thus not used in computations

Parameters

		Value	Standard Error
C	Intercept	-9,28165E-4	9,92482E-4
	A	0,47139	0,00256
	B	0,53418	0,00265

Statistics

	C
Number of Points	6
Degrees of Freedom	3
Residual Sum of Squares	8,75808E-6
Adj. R-Square	0,99991

Summary

	Intercept		A		B		Statistics
	Value	Standard Error	Value	Standard Error	Value	Standard Error	Adj. R-Square
C	-9,28165E-4	9,92482E-4	0,47139	0,00256	0,53418	0,00265	0,99991

ANOVA

		DF	Sum of Squares	Mean Square	F Value	Prob>F
C	Model	2	0,16495	0,08248	28251,74893	3,86843E-7
	Error	3	8,75808E-6	2,91936E-6		
	Total	5	0,16496			

Residual vs. Independent Plot

	ISOTOP PATTERN			NORMALIZED VALUES				OBTAINED			CALCULATED ISOTOP PATTERNS		
m/z	H	D	LC MS	NH	ND	NLC		PARAMETERS			HA	DB	HA+DB
325,2	25689	379	58995	0,789435	0,004113	0,372997		A	0,47139		0,372132	0,002197	0,374329
326,2	6610	1149	16299	0,203128	0,012469	0,103051		B	0,53418		0,095753	0,006661	0,102413
327,2	192	71240	65685	0,0059	0,773087	0,415294		R^2	0,99991		0,002781	0,412968	0,415749
328,2	50	17006	15246	0,001537	0,184547	0,096393					0,000724	0,098581	0,099306
329,2	0	2198	1741	0	0,023852	0,011007					0	0,012741	0,012741
330,2	0	178	199	0	0,001932	0,001258					0	0,001032	0,001032
	32541	92150	158165										

Fig. S29 The results of computational analysis of LC-MS analysis of winter windscreen washer fluid with addition of the deuterated denatonium benzoate standard

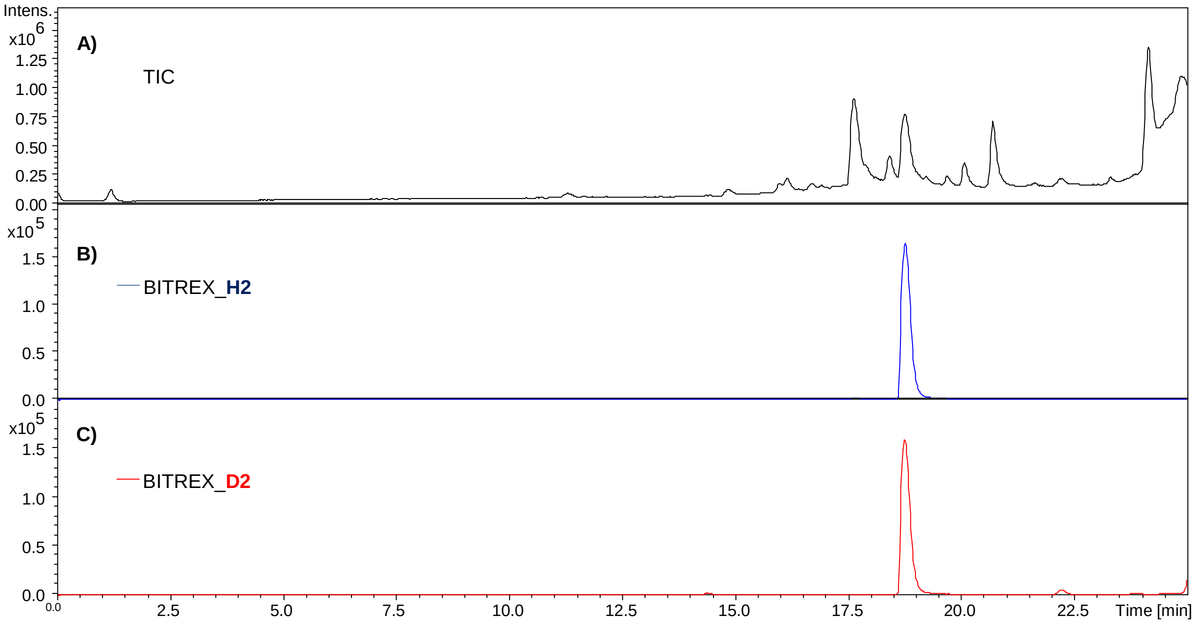

Fig. S30 The LC-MS analysis of commercially available winter windscreen washer fluid. Total ion chromatogram (A) and extracted ion chromatograms of detected denatonium cataion (B) and added deuterated standard (C)

Multiple Regression (2015-05-11 10:53:06)

- Notes
- Input Data
- Masked Data - Values Excluded from Computations
- Bad Data (missing values) -- Values that are invalid and thus not used in computations
- Parameters

		Value	Standard Error
C	Intercept	-0,0025	0,00515
	A	0,36427	0,01494
	B	0,65072	0,01328

Statistics

	C
Number of Points	6
Degrees of Freedom	3
Residual Sum of Squares	2,34111E-4
Adj. R-Square	0,99807

Summary

	Intercept		A		B		Statistics
	Value	Standard Error	Value	Standard Error	Value	Standard Error	Adj. R-Square
C	-0,0025	0,00515	0,36427	0,01494	0,65072	0,01328	0,99807

ANOVA

		DF	Sum of Squares	Mean Square	F Value	Prob>F
C	Model	2	0,20227	0,10113	1295,96107	3,93093E-5
	Error	3	2,34111E-4	7,80369E-5		
	Total	5	0,2025			

Residual vs. Independent Plot

	ISOTOP PATTERN			NORMALIZED VALUES				OBTAINED			CALCULATED ISOTOP PATTERNS		
m/z	H	D	LC MS	NH	ND	NLC		PARAMETERS			HA	DB	HA+DB
325,2	3246	379	41048	0,697614	0,004113	0,258079		A	0,36427		0,25412	0,0026763	0,256796
326,2	936	1149	10475	0,201161	0,012469	0,065859		B	0,65072		0,0732768	0,0081137	0,08139
327,2	397	71240	84344	0,085321	0,773087	0,530292		R^2	0,99807		0,03108	0,5030634	0,534143
328,2	74	17006	20608	0,015904	0,184547	0,129568					0,0057932	0,1200884	0,125882
329,2	0	2198	2387	0	0,023852	0,015008					0	0,0155212	0,015521
330,2	0	178	190	0	0,001932	0,001195					0	0,001257	0,001257
	4653	92150	159052										

Fig. S31 The results of computational analysis of LC-MS analysis of winter windscreen washer fluid with addition of the deuterated denatonium benzoate standard

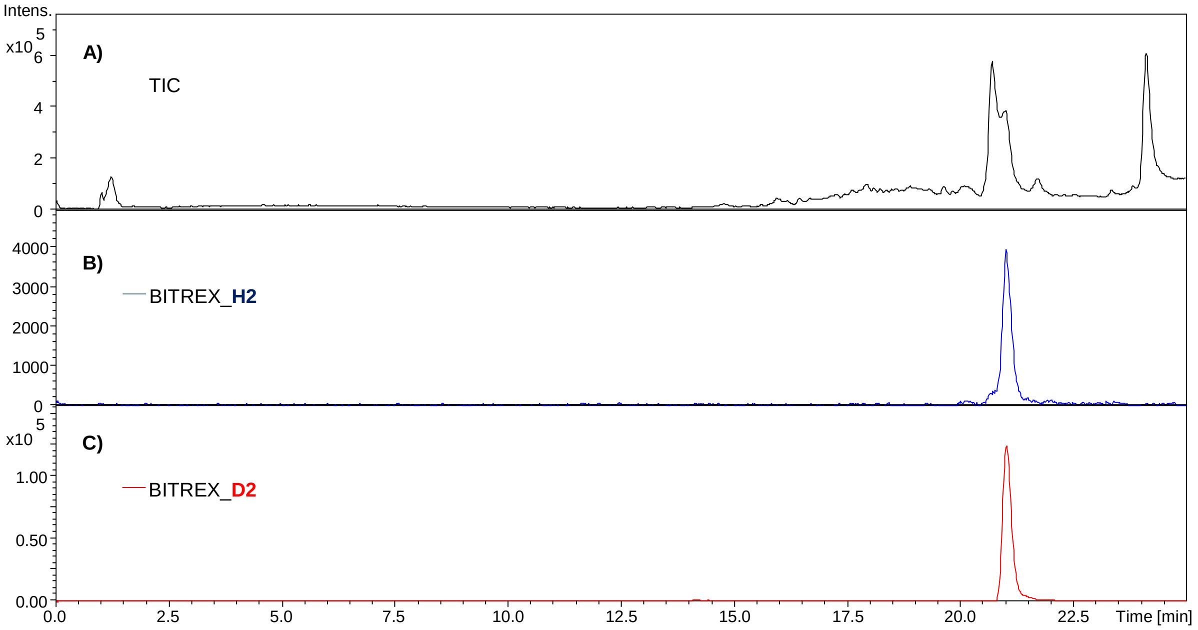

Fig. S32 The LC-MS analysis of commercially available summer windscreen washer fluid. Total ion chromatogram (A) and extracted ion chromatograms of detected denatonium cataion (B) and added deuterated standard (C)

Multiple Regression (2015-05-11 10:55:54)

- Notes
- Input Data
- Masked Data - Values Excluded from Computations
- Bad Data (missing values) -- Values that are invalid and thus not used in computations
- Parameters

		Value	Standard Error
C	Intercept	0,00287	0,00143
	A	0,03322	0,00386
	B	0,94954	0,00377

- Statistics

	C
Number of Points	6
Degrees of Freedom	3
Residual Sum of Squares	1,79793E-5
Adj. R-Square	0,99993

- Summary

	Intercept		A		B		Statistics
	Value	Standard Error	Value	Standard Error	Value	Standard Error	Adj. R-Square
C	0,00287	0,00143	0,03322	0,00386	0,94954	0,00377	0,99993

- ANOVA

		DF	Sum of Squares	Mean Square	F Value	Prob>F
C	Model	2	0,41174	0,20587	34351,55399	2,88529E-7
	Error	3	1,79793E-5	5,9931E-6		
	Total	5	0,41176			

- Residual vs. Independent Plot

	ISOTOP PATTERN			NORMALIZED VALUES				OBTAINED			CALCULATED ISOTOP PATTERNS		
m/z	H	D	LC MS	NH	ND	NLC		PARAMETERS			HA	DB	HA+DB
325,2	1554	379	2020	0,748195	0,004113	0,032362		A	0,03322		0,024855	0,0039053	0,0287603
326,2	461	1149	1222	0,221955	0,012469	0,019578		B	0,94954		0,007373	0,0118396	0,019213
327,2	60	71240	46083	0,028888	0,773087	0,738297		R^2	0,99993		0,00096	0,7340774	0,735037
328,2	2	17006	11006	0,000963	0,184547	0,176327					3,2E-05	0,1752347	0,1752667
329,2	0	2198	1764	0	0,023852	0,028261					0	0,0226488	0,0226488
330,2	0	178	323	0	0,001932	0,005175					0	0,0018342	0,0018342
	2077	92150	62418										

Fig. 33S The results of computational analysis of LC-MS analysis of summer windscreen washer fluid with addition of the deuterated denatonium benzoate standard

References

1. Mikkelsen K, Nielsen SO (1960) Acidity measurements with the glass electrode in H_2O-D_2O mixtures. J Phys Chem 64:632-637

2. Mirgorodskaya OA, Kozmin YP, Titov MI, Körner R, Sönksen CP, Roepstorff P (2000) Quantitation of peptides and proteins by matrixassisted laser desorption/ionization mass spectrometry using ^{18}O-labeled internal standards. Rapid Commun Mass Spectrom 14:1226-1232

3. Smith MB, March J (2007) March`s Advanced Organic Chemistry: reactions, Mechanisms, Structure, 6th ed.; Wiley-Interscience, Chapter 18, pp1621-1623

4. Ng L-K, Hupe M, Harnois J, Lawrence AH (1998) Direct injection gas chromatographic/mass spectrometric analysis for denatonium benzoate in specific denatured alcohol formulations. Anal Chem 70:4389-4393
